# Soil contamination assessment of a 19th-century abandoned Sb–Au mine in northern Portugal

**DOI:** 10.1007/s10653-026-03359-6

**Published:** 2026-07-20

**Authors:** Giulia Resta, Patrícia Santos, Marcus Monteiro, Rui Melo, Morgana Carvalho, Ana Carvalho, Alexandre Lima, Eric Font, José A. Ribeiro, Manuel Azenha, Thierry Adatte, Deolinda Flores

**Affiliations:** 1 Departamento de Geociências Ambiente e Ordenamento do Território, Faculdade de Ciências, Instituto de Ciências da Terra, Universidade do Porto, Rua do Campo Alegre S/N, 4169-007 Porto, Portugal; 2 Departamento de Química e Bioquímica, Faculdade de Ciências, CIQUP/IMS, Universidade do Porto, Rua do Campo Alegre 687, 4169-007 Porto, Portugal; 3 Departamento de Ciências da Terra, Faculdade de Ciências e Tecnologia, Universidade de Coimbra, 3030-790 Coimbra, Portugal; 4 INESCTEC–Centro de robótica e sistemas autónomos, Instituto Superior de Engenharia do Porto, 4249-015 Porto, Portugal; 5 Department of Earth Sciences, University of Coimbra, Institute Dom Luiz, Coimbra, Portugal; 6 Institute of Earth Sciences (ISTE), University of Lausanne, Lausanne, Switzerland

**Keywords:** Contamination pathways, Potentially toxic elements, Hg mobility, Mining residues

## Abstract

Abandoned mines are among the main sources of long-term soil contamination, often leaving behind persistent concentrations of potentially toxic elements (PTE) that pose environmental and health risks. Ribeiro da Serra Sb–Au mine, in Portugal, active from 1858 to 1890, has left a significant environmental legacy. This study mapped the spatial distribution of mine processing residues, elemental characterisation distinguishing between anthropogenic and natural enrichment by determining soil sample concentrations of PTE, Hg mobility, and Total Organic Carbon (TOC) quantification. Multivariate analysis, spatial interpolation and comparison with Enrichment Factor were employed with the aim of understanding the distributions and sources of PTEs in the soils. Mercury is still present at the site, revealing high mobile (18.72 mg kg^−1^) and semi-mobile (3.58 mg kg^−1^) concentrations accumulated in waste piles, a remnant of Au amalgamation processes. High Hg concentrations (20.75 mg kg^−1^) pose significant environmental risks, even after more than a century, such as bioaccumulation potential and soil toxicity. Also, there are high concentrations of Pb (449.03 mg kg^−1^) in the waste piles from the Sb processing. This study highlights the critical importance of interpreting natural enrichment values of elements of Hg (EF = 267) and Pb (EF = 18) to discern pollution pathways resulting from mining and processing activities. Interpretation of natural enrichment values leads to a more accurate evaluation of environmental contamination and its sources. The Enrichment Factor shows that Sb (EF = 133) and Hg (EF = 267) are the elements that present extremely high enrichment (EF > 40).

**Supplementary Information:**

The online version contains supplementary material available at https://doi.org/10.1007/s10653-026-03359-6.

## Introduction

During the 19th and 20th centuries, mining operations worldwide largely ignored environmental considerations, leaving waste piles and industrial structures abandoned without any mitigation measures. Large volumes of mining residues produced were accumulated in waste piles and act as a source for the accumulation and mobilisation of PTE. These unmanaged residues continue to release contaminants capable of degrading soil, water, and air quality, posing risks to ecosystems and human health (eg. Barago et al., 2025; Moyo et al., 2023; Sigué et al., 2025, Konanç et al., 2024).

Portugal was no exception, and different studies have been conducted in abandoned mines, mainly focusing on identifying contamination, acid mine drainage, general risk patterns and remediation (eg Mourinha et al., 2022; Carvalho, 2017, Ferreira da Silva, 2013, Antunes et al., 2016, 2018, Candeias et al., 2015, Sant’Ovaia et al., 2023, Durães et al., 2021, Barroso et al., 2025, Gomes, 2025, Valente et al., 2009, Reis et al., (2010), Monteiro et al., (2024,2025 )).

The present study focuses on the study of soil contamination surrounding a northern Portugal Sb–Au mine, Ribeiro da Serra active between 1858 and 1890, and that was once one of the most important Sb–Au mines of the Dúrico-Beirão mining district.

According to historical data of the mine, besides the mineralogical composition of the ores, there were different chemical components added in the ore processing (Carvalho, 1969). The stibnite (Sb_2_S_3_) concentration was achieved after mechanical processes, flotation and fusion. Mercury amalgams were also used in Ribeiro da Serra Mine washing plants to recover gold, (approximately 20 g of Hg for each gram of Au produced). The Au-Hg amalgam was burnt, concentrating the gold into a pellet and releasing large quantities of elemental mercury (Hg_0_) into the air (Paruchuri et al., 2010; Telmer & Stapper, 2007; UNEP, 2012) and remaining mining residues were disposed in the existing waste piles.

Mercury is a potentially harmful trace element in the environment and one of the World Health Organisation’s leading chemicals of concern (O’Connor et al., 2019). According to Kabata-Pendias and Mukherjee (2007), mercury has a low abundance in the Earth’s crust (0.02–0.09 mg kg^−1^), where the highest levels of mercury appear associated with organic soils (histosoils) since its retention in the environment could be maximised by the quantity of organic carbon present in the biomass of the topsoils. However, most topsoils hold considerable amounts of this metal, especially close to mining and smelting areas, since this element can be leached and drained from the washing/concentration plant and waste piles.

The assessment of different concentrations of PTE in the mine surrounding soils can be used as an indicator for contamination; these elements are persistent, non-biodegradable and can accumulate in soils and sediments through adsorption and co-precipitation (Djibril, 2026; Yiika et al., 2023). Data processing combining multivariate statistical techniques and spatial distribution analysis has been widely used to identify potential contaminant sources, allowing the distinction between natural geogenic and anthropogenic contributions through the interpretation of geochemical associations and spatial patterns (Hou et al., 2017; Shi et al., 2024).

Different contamination indexes can be applied to distinguish between natural background values and anthropogenic inputs, as they compare measured concentrations in soils and sediments with geochemical baseline levels or reference elements, thereby quantifying the degree of enrichment above natural variability. Elevated index values indicate that contaminant concentrations exceed expected natural levels and may therefore reflect anthropogenic contributions such as mining, industrial activities, agriculture, or urbanisation (Soetan et al., 2024; Swain, 2024). Moreover, the contamination and the anthropogenic effects caused by the mining process cannot be carried out straightforwardly since the soils in mining areas were already naturally enriched in metal(oid)s due to the geological processes that gave origin to mineral deposits (Sant’Ovaia et al., 2023; Santos et al., 2023).

Sequential extraction techniques may contribute to the identification of defined Hg species by partitioning mercury among different geochemical fractions associated with varying binding strengths and environmental reactivities. This approach provides valuable information on the origin of Hg, distinguishing between geogenic and anthropogenic sources, and allows the assessment of its potential mobility, bioavailability, and environmental risk (Eckley et al., 2020; Monteiro et al., 2024).

The objectives of this study consist of (I) assessing the environmental legacy of the Sb–Au mining activities, with particular emphasis on how past exploitation has shaped present-day soil contamination patterns and the persistence of mining-derived contaminants in the surrounding soils; (II) study the concentration of mercury, its distribution and mobility/bioavailability, given its high toxicity, long-range transport, and relevance to both environmental and public-health concerns, and considering that previous studies from this area were focused in potentially toxic elements that were associated with mineral paragenesis and did not included Hg determinations. The study intends to provide a more accurate assessment of pollution pathways, enabling future evidence-based strategies for environmental management and remediation.

The main added value of this study is therefore to provide the first integrated assessment of Sb–Hg–As contamination and Hg mobility in a Sb–Au mine from Dúrico-Beirão mining district, using different methods (geochemical characterisation, multivariate analysis, spatial interpolation, Enrichment Factor, sequential extraction procedures) and emphasising the careful interpretation of “natural enrichment” in a highly mineralised geological context. This fills a gap regarding studies known in this type of deposit in Portugal.

## Geological setting

The Ribeiro da Serra mine site is situated in the Iberian Massif of Northern Portugal, within a Proterozoic or Paleozoic unit that constitutes the occidental side of the Central Iberian Zone. Ribeiro da Serra mine is included in the Dúrico-Beirão mining district, known for over a dozen deposits, oriented NW-SE along 90 km (Couto, 1993). It is estimated that this district produced ~ 1200 T of antimony and 2 T of gold, partially recovered in the Ribeiro da Serra and Tapada (also abandoned) mines (Couto et al., 1990).

The study area is located on the western limb of the Valongo’s anticline. within a metallogenic corridor that includes Sb–Au mineralisations and other ore occurrences such as Pb–Zn–Ag, W–Sn, and coal. Sb–Au mineralisations are concentrated on the inverse flank between the Covelo and Sobrido (abandoned mines) regions. Sb–Au deposits are hosted in pre-Ordovician country metasedimentary rocks from Beiras Group, and breccias from the lower Carboniferous (Couto, 1993). The most productive veins in Ribeiro da Serra occur in the N–S direction, also dipping to W (Carvalho, 1969). Neiva et al. (2008) identified stibnite (Sb_2_S_3_), berthierite (FeSb_2_S_4_), arsenopyrite (FeAsS), pyrite (FeS_2_) and plagigonite (Pb_5_Sb_8_S_17_), as major to locally abundant minerals in the mine paragenesis. Gold in these veins was associated with arsenopyrite (FeAsS), and electrum (naturally occurring gold-silver alloy) associated with jamesonite (Pb_4_FeSb_6_S_14_) (Couto et al., 1990; Neiva et al., 2008).

This geological setting can explain the natural enrichment of As, Sb, and Pb, and supports the background values adopted in the study by indicating that elevated concentrations may reflect local geogenic inputs rather than only mining contamination.

## Materials and methods

**Fig. 1:**
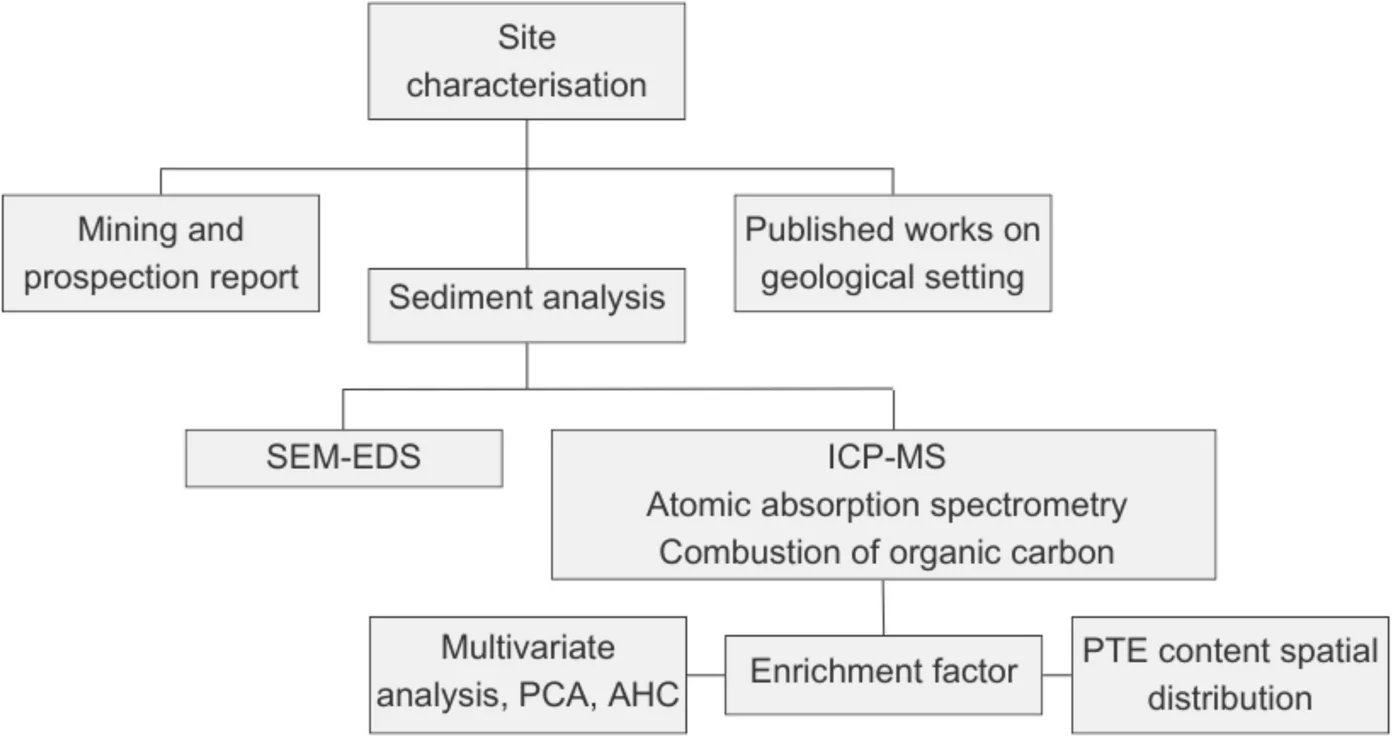
Flowchart regarding methodologies used in the soil characterisation surrounding Ribeiro da Serra Mine (SEM-EDS, scanning electron microscopy–energy dispersive spectroscopy; ICP-MS, inductively coupled plasma mass spectrometry; PCA, principal component analysis; AHC, agglomerative hierarchical clustering; PTE, potentially toxic element)

To better understand the distribution of Hg and other PTEs in the area, geochemical data, multivariate analysis, and the spatial interpolation of the PTE distribution were used to study the relationships between geological features, the mine structures and the mine drainage area (Fig. 1).

Figure 3 summarises the methodology applied in the study. A batch of 54 samples was selected from a total of 157 samples previously collected from the study area in the scope of the AUREOLE Project (doi.org/https://doi.org/10.54499/ERA-MIN/0005/2018), ensuring a representative analytical dataset while remaining within the financial limitations imposed. This strategy allowed the study to preserve spatial and lithological coverage without compromising the feasibility of geochemical analyses.

These 54 samples were analysed by Inductively Coupled Plasma Mass Spectrometry (ICP-MS) for elemental analysis, while mercury content was determined through Atomic Absorption Spectrometry (AAS). Finally, 12 samples were assayed using the Environmental Protection Agency (EPA) 3200 sequential leaching procedure. Basic and multivariate statistics were also employed to understand elemental relationships in the area. Principal Component Analysis (PCA) was used to identify chemical element distribution patterns, and Agglomerative Hierarchical Clustering (AHC) to describe visually the association between the PTE.

Spatial interpolation allowed the analysis of the spatial distribution patterns of PTE values.

The Enrichment Factor (EF) was also calculated as part of the environmental assessment regarding 10 background samples collected uphill the mine in soils that were not affected by the mine.

### Study area

**Fig. 2:**
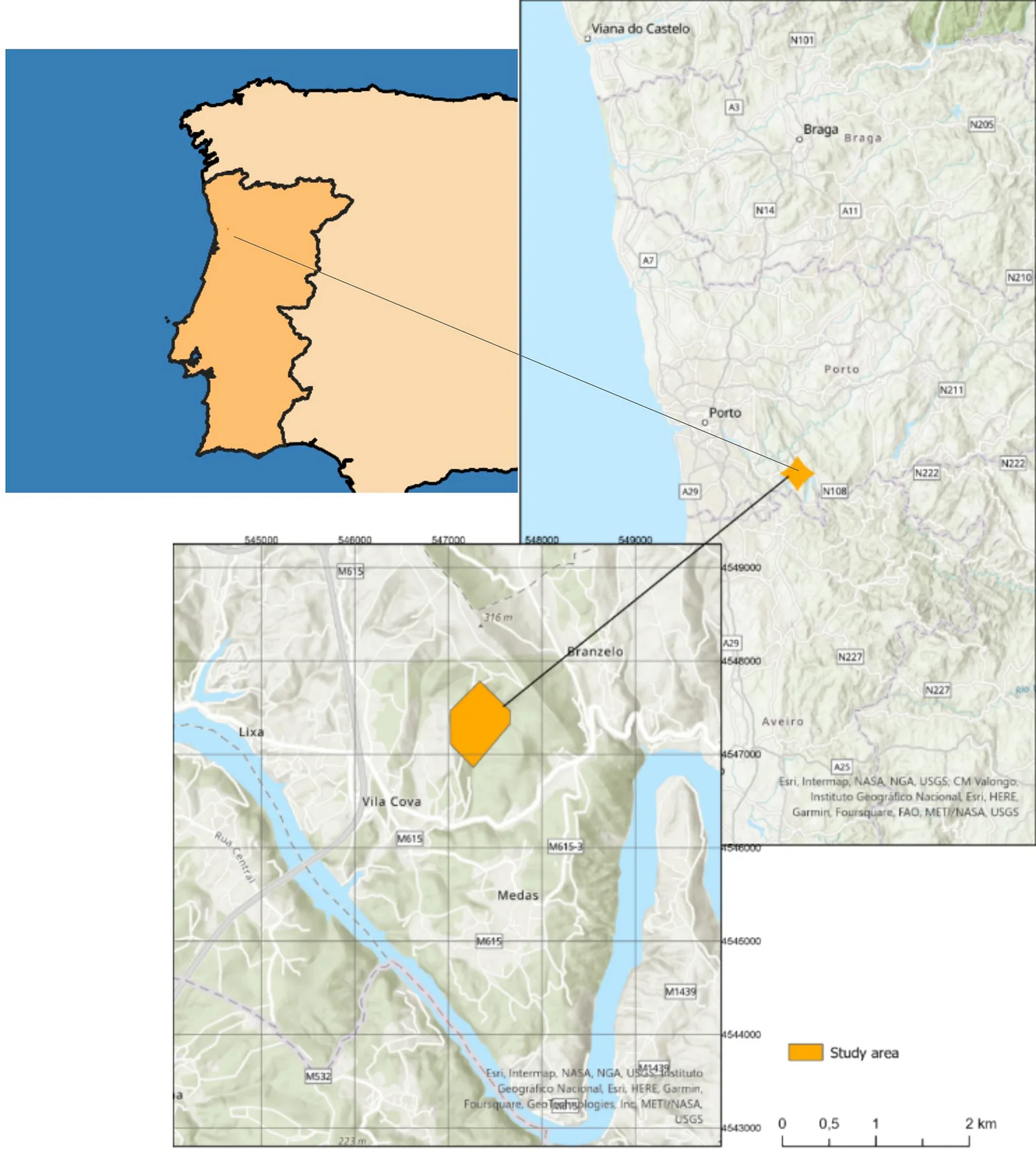
The study area located in the abandoned in and around the mining works of Ribeiro da Serra; coordinates in UTM 29N (WGS84)

The study area corresponds to an abandoned Sb–Au mining site in northern Portugal, which covers approximately 34 hectares (UTM zone 29N, 547,390.71 m E, 4,547,418.61 m N) (Fig. 2). Known as the Ribeiro da Serra concession, it lies within the Gondomar municipality, approximately 15 km from Porto.

The abandoned mine facilities are located within a waste pile area on the right flank of a drainage valley where a water stream may seldom flow when rainfall is exceptionally high. The largest waste pile (~ 2 ha) is located next to the mining works of Ribeiro da Serra.

**Fig. 3:**
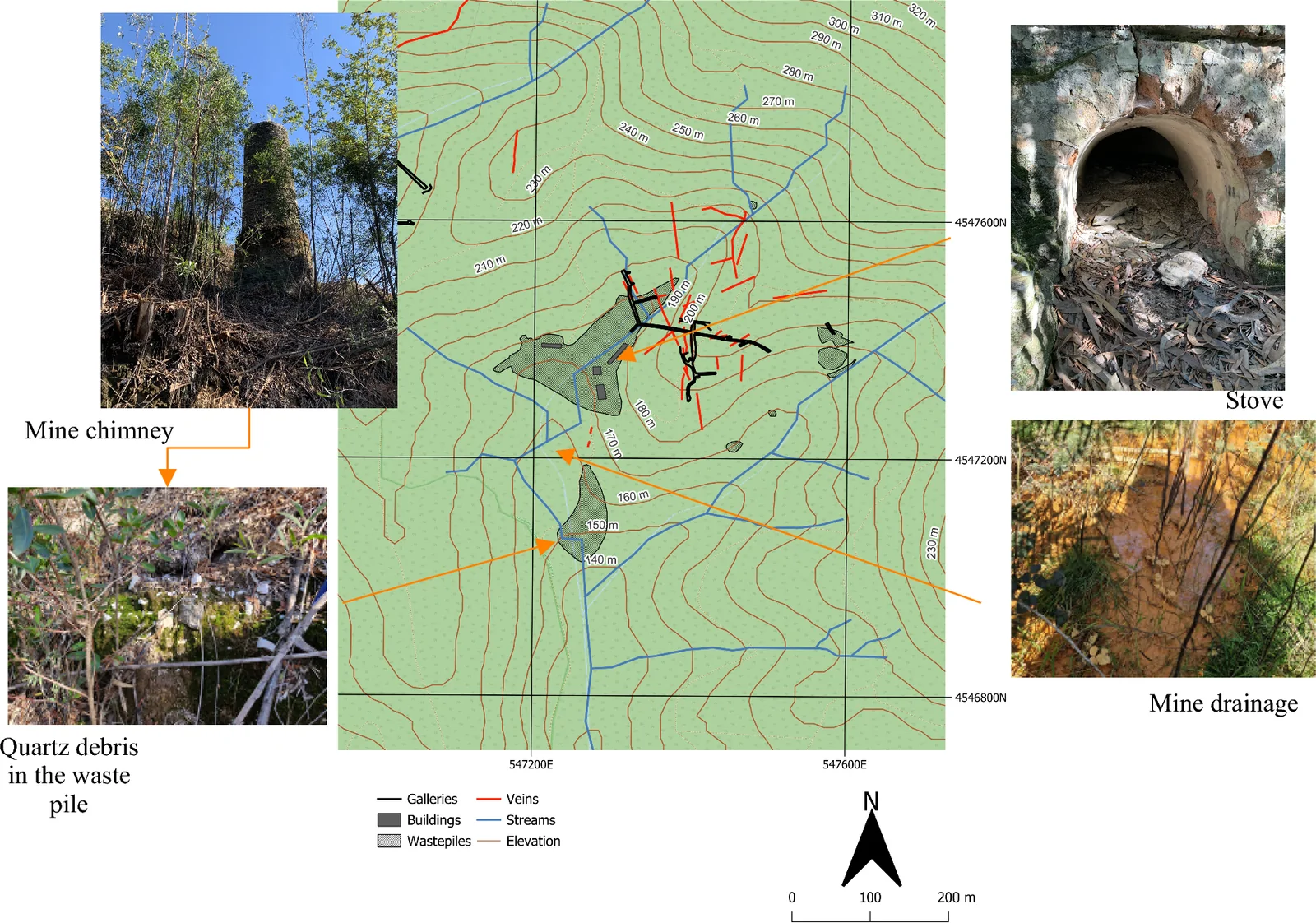
Overview of Ribeiro da Serra area (adapted from (Kernow Mining Portugal, 2009); coordinates in UTM 29N (WGS84)

The second relevant waste pile (0.60 ha) is located at the southernmost part of the study area and inside the drainage valley. Both waste piles cover ~ 7% of the study area. Figure 3 shows the location of a structure that could be a ventilation shaft in the northern waste pile; visual evidence of the mine drainage with oxidised deposits in orange and dispersed white quartz debris next to a chimney in the second waste pile. The features related to the mine presented in Fig. 2 are obtained from a report by Kernow Mining Portugal (2008).

### Soil sampling

**Fig. 4:**
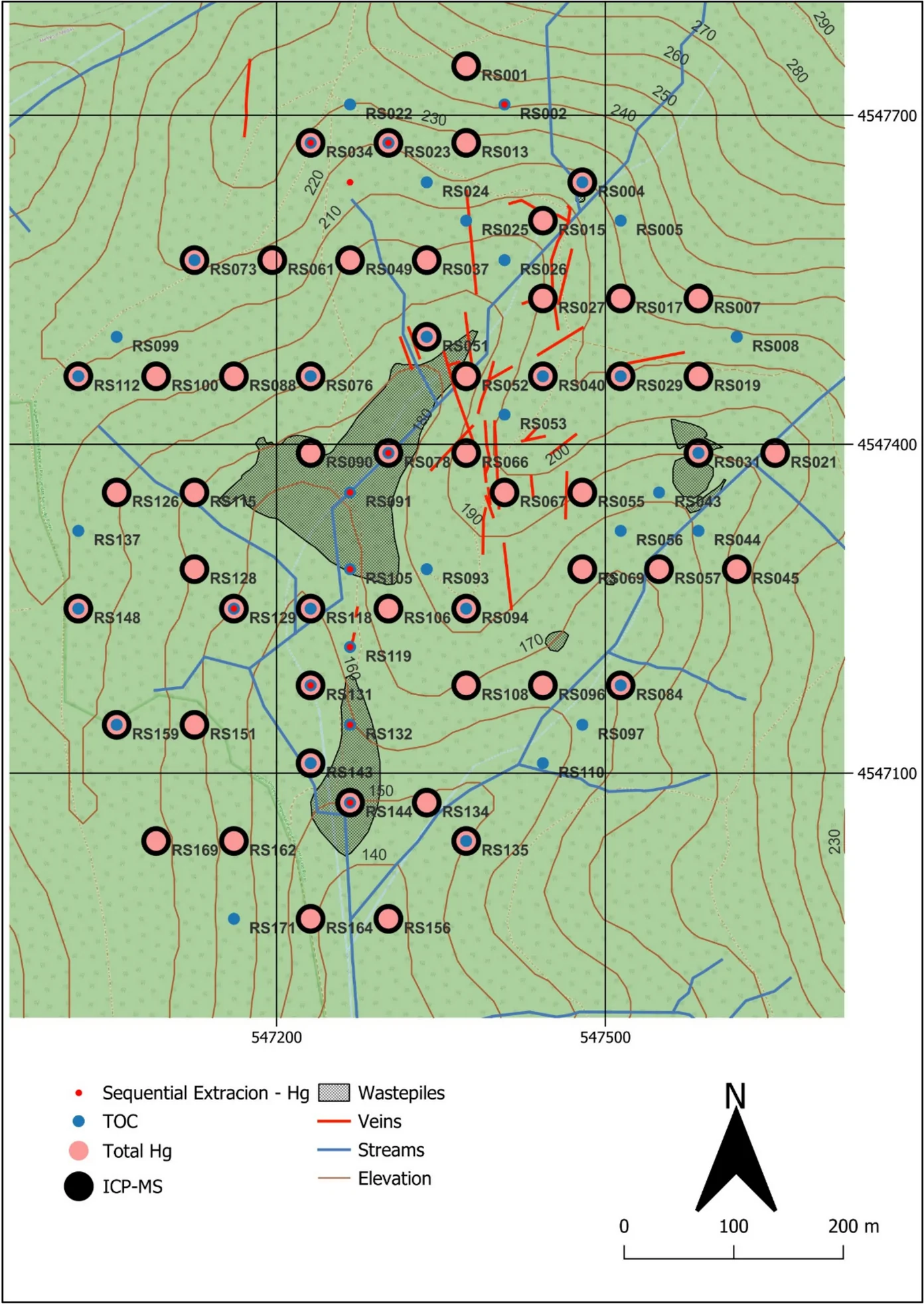
Sediment sampling grid selected by each methodology; coordinates in UTM 29N (WGS 84)

The present study used a selection of sediment (topsoil and mining waste residues) samples previously collected in the scope of Project AUREOLE. The sampling grid also included background samples and was oriented 45° from the geographic north (perpendicular to the orientation of the Dúrico-Beirão mining district). Each sample is spaced 50 m from the other (Fig. 4). the geographic coordinates and the land cover of each sampling site were recorded. During sampling, branches, weeds and stones were removed. The depth of the samples collected with a clean and non-oxidised scoop is around 15 to 30 cm and ~ 500 g of sediment was collected for each sample. The samples were stored in polyethylene zip-lock bags and later dried in a stove under 35–40 °C.

### Soil geochemical characterisation

Bureau Veritas laboratory in Vancouver (Canada) analysed the sediment samples by ICP-MS, using Agilent 7850 ICP mass spectrometer. The analysis consisted of the concentrations of Hg, Sb, As, Ba, Cd, Pb, Co, Cu, Cr, Mo, Ni, Ag, Se, Tl, U, V and Zn. A subset of 54 samples was selected from the initial 157 to ensure a representative analytical dataset while remaining within the financial limitations imposed by the high cost of laboratory determinations. This strategy allowed the study to preserve spatial and lithological coverage without compromising the feasibility of geochemical analyses. The samples were pre-processed, sieved at 2 mm, milled to less than 200 µm in an agate mortar and homogenised mechanically.

Samples were digested by multi-acid, heated 0.25 g split in HNO_3_, HClO_4,_ and HF to fuming and taken to dryness. The quality control method involved the certified reference materials (STD OREAS45H, STD OREAS501D, STD OREAS25A-4A), blanks, and random duplicates.

Pulp duplicate samples show excellent reproducibility, with most relative percentage differences (RPD) below 5% and only minor deviations approaching 10%, indicating low analytical variability. Certified reference materials (OREAS standards) display consistent results and broadly align with expected values, suggesting acceptable analytical accuracy.

The analysis of total mercury in soils was performed by Atomic Absorption Spectrometry at the Laboratory of Palaeomagnetism of the Department of Earth Sciences at the University of Coimbra. The equipment comprised the Lumex RA-915 Lab. It measured the mercury content of 114 sub-samples using 5 g, milled in an agate mortar under 200 µm. The configuration includes a high-frequency atomic absorption spectrometer set at Mode 1 (750–800 °C), specific for soil and sediments. This mode has a flow rate of 1 L min^−1^ and a duration of 200 s. All measurements include duplicates and a certified standard NCS DC 73309, to define the R-value for calibration.

Most samples exhibited an RSD of less than 5%, meeting the acceptance criteria normally applied to instrumental methods. Only six samples exhibited an RSD above 7%, a value justifiable by low concentration or intrinsic variability of the samples. The certified standard NCS DC 73309, included in the series of measurements, presented a mean value of 75.60 µg kg^−1^ with an RSD of 1.73%, confirming the instrumental stability and the suitability of the method for quantification.

In some areas of interest within the area affected by mine drainage, petrographic and geochemical analysis by Scanning Electron Microscopy with Energy Dispersive X-ray Spectroscopy (SEM-EDS) was conducted in Centro de Materiais da Universidade do Porto (CEMUP). A total of four samples were analysed by a FEI QUANTA 400 FEG ESEM, a high-resolution field emission scanning microscope equipped with EDS (Edax Genesis X4M) for point analysis or chemical element mapping.

### Sequential extraction of mercury

The sequential extraction technique is used to determine heavy metal species related to geochemical phases in soils, sediments and waste piles. This method provides important insight as concerns various fractions in which the heavy metal occurs as potential toxicity levels (Yika et al., 2023).

A Sequential Extraction Procedure (SEP) method was applied to non-milled samples but manually sieved at 2 mm to remove roots and weeds. The SEP was executed according to the method proposed by the US Environmental Protection Agency (EPA) 3200 (EPA, 2014) procedure to fractionate the Hg from the soils in three fractions: mobile, semi-mobile, and non-mobile (Table 1). The Hg present in the extracts was quantified by Cold Vapour Atomic Absorption Spectroscopy (CV-AAS). The Thermo Scientific iCE 3000 Series double-beam atomic absorption spectrometer was used, combined with the VP100 accessory, a continuous flow vapor generation system. The calibration curves were done with standard solutions prepared in 10% HCl (w/v) in the concentration range of 2–20 µg L^−1^. The total measurement time for each sample was 100 s and the Hg cold vapor was generated by aspirating the sample solution (at a flow rate of 7.5 mL min^−1^) for 25 s and mixing it under continuous flow with solutions of (i) NaBH_4_ 1% (w/v) stabilized in NaOH 0.5% (w/v) (flow rate of 1.6 mL min^−1^) and (ii) HCl 10% (w/v) (flow rate 0.7 mL min^−1^). The Hg vapour was carried to the absorption cell by a N2 flow (at a flow rate of 100 mL min^−1^), where the absorption was measured at 257.3 nm. Some sediment samples were, submitted to the SEP procedure in duplicate, and the Hg content in extracts was analysed to estimate the uncertainty affecting the experimental results.

**Table 1:** Summary of EPA 3200 sequential extraction methodology, operationally defined fractions and related extracted species

Step	Reagents	Experimental conditions	Hg species	Fraction
1	HCl 2% + EtOH 10%	Ultrasound-assisted extraction for 7 min at 60 °C (3 times)	CH_3_HgCl; CH_3_CH_2_HgCl; HgCl_2_; Hg(OH)_2_; Hg(NO_3_)_2_; HgSO_4_; HgO; Hg^2+^ complex	Mobile
2	1:2 HNO_3_	Extraction for 20 min on a water bath at 95 °C (2 times)	Hg^0^; Hg^0^–Metal (amalgam); Hg^2+^ complex; Hg_2_Cl_2_ (minor)	Semi-mobile
3	1:6:7 HCl:HNO_3_: H_2_O	Extraction for 20 min on a water bath at 95 °C (2 times)	Hg_2_Cl_2_ (major); HgS; HgSe	Non-mobile
4	*Aqua regia*	USEPA 3051A	Strongly bound minerals	Residual

Table 1 provides the operationally defined Hg fractions and individual species that may be determined by the EPA 3200 procedure for sequential extraction of Hg from the soil matrix. In short, the mobile fraction includes some of the most toxic Hg species (alkyl mercury species; [CH_3_Hg]^+^, etc.) and the most labile and bioavailable Hg species (soluble inorganic species; (HgCl_2_), (Hg(OH)_2_, etc.) with high potential to groundwater contamination and soil-to-plant transfer assessment. The semi-mobile fraction includes mainly elemental mercury (Hg^0^) and Hg^0^-metal amalgams that are not readily bioavailable. The non-mobile mercury fraction contains chemically stable mercury species in the soil over geologic times (like HgS) and, thus, the least environmentally concerning mercury species (Han et al., 2003; Reis et al., 2016).

### Total organic carbon

The 44 non-milled samples were manually sieved to remove roots and weeds for Total Organic Carbon (TOC) analysis. Rock-Eval analyses were performed at the Institute of Earth Sciences of the University of Lausanne (Switzerland) using a Rock-Eval 6 (Vinci Technologies). Rock-Eval analysis is based on continuous measurement of effluents (HC–hydrocarbon, CO and CO_2_) released during the thermal cracking of organic compounds (and thermal decomposition of carbonates) in pyrolytic conditions (up to 650 °C in an inert atmosphere), then during the combustion of residual organic and inorganic carbon (up to 850 °C in an oxidative atmosphere). A flame ionisation detector identifies the release of HC during the pyrolytic stage, while an infrared cell detects the release of CO and CO_2_ during both stages. The resulting thermograms are used to calculate standard parameters by integrating the amounts of HC, CO and CO_2_ between defined temperature limits. The release of CO and CO_2_ was monitored and detected as peaks at different temperatures as follows: S1 peak was acquired from 25 to 300 °C and represents the amount of free and adsorbed hydrocarbons already present in the sample. The S2 peak was obtained from 300 to 500 °C and corresponds to the hydrocarbons generated directly from the kerogen. The S3 peak was monitored from 300 to 390 °C and represents the amount of CO and CO_2_ generated from the pyrolysis of oxygen-containing compounds present in the kerogen. Measurements were calibrated using the IFP 160000 standard. The concentrations of total organic carbon (TOC) and mineral carbon (MinC) are characterised by uncertainties of ± 0.01 wt% based on the IFP 160000 calibration standard (Vinci Technologies, France) having total organic carbon of 3.28 ± 0.14 wt%.Rock-Eval pyrolysis provides parameters such as the total organic-carbon content (TOC, %), mineral carbon (MinC, %), hydrogen index (HI, mg HC/g TOC, HC = hydrocarbons), oxygen index (OI, mg CO2/g TOC) and Tmax (°C). HI, OI and Tmax values that provide an overall measure of the type and maturation of the organic matter (Behar et al., 2001; Espitalié et al., 1985) were interpreted for TOC ≥ 0.2% and S2 values ≥ 0.2 mg HC/g.

### Statistical analysis

The pre-statistical treatment included the Shapiro-Wilk normal distribution verification for each PTE, conducted in XLSTAT (v.2022.4.1). The data analysis also included Spearman correlation and Principal Component Analysis (PCA) using the software ioGAS (v.7.4) to define the source apportionment of metals.

Beforehand, the data was transformed using the Centred Log-Ratio (CLR) to remove false correlations on ioGAS. The CLR was processed using compositional data analysis (Aitchison, 1986). In this transformation, each variable is divided by the geometric mean of all values and then log normalised (centred).

Principal Component Analysis can help find patterns of chemical element data that can assist the identification of contaminants’ origin. It reduces a data set holding a larger number of variables by finding a linear combination of the original variables. The samples were separated into two groups: the background area (from uphill) and the mining area of influence to study the patterns in the PCA biplot.

The Agglomerative Hierarchical Clustering (AHC) provides a dendrogram that visually describes the association between the potentially toxic elements (PTE) by seeking the hierarchy of clusters from the multielement concentration values by seeking similarities and dissimilarities between them. The method was conducted using XLSTAT (v.2022.4.1) software and corresponded to the Euclidean distance for the proximity type and Ward’s for the agglomeration method.

As suggested by Sigué et al. 2025, there is a constant and supportive link between the conclusions drawn from the PCA and AHC analyses. In addition to PCA and AHC improve our understanding of the relationship that affects the distribution of PTEs in the study area.

### Background and reference values

The concentrations determined in soils for the multiple metals and metalloids were compared with the Portuguese Environmental Agency (APA, 2019) reference to Table C for agriculture soil use for shallow soils as these values are for legal practices and recommendations in Portugal. In addition, natural background concentrations vary greatly on different geological characteristics. Thus, the first Geochemical Atlas of Portugal (Inácio et al., 2008) was used as a national parameter.

The local geochemical background was also determined by removing the outliers and by later calculating the median for each element from 10 samples located uphill the mine (RS001. RS007, RS023, RS034, RS037, RS045, RS073, RS084, RS100, RS126).

### Environmental assessment

The Enrichment Factor index emphasises the shared contributions of anthropogenic and geogenic sources contributing to the presence of metals in the study area (Afahnwie et al., 2025). It normalises one metal concentration in the topsoil according to the concentration of a reference element, which is a stable element in the soil (frequently Fe or Al), characterised by the absence of vertical mobility and degradation phenomena (Barbieri, 2016). In this case, Al was preferred as normalising element in the assessment of anthropogenic change, as the soil was rich in clay and aluminosilicate content. Fe may be less ideal due to its presence in mining residues, as well as it can also show alterations caused by diagenetic processes (Birch, 2020).

Enrichment Factor (EF) levels are calculated by the formula below:

$$EF = {{\left( {\left[ {Al} \right]background \times \left[ x \right]} \right)} \mathord{\left/ {\vphantom {{\left( {\left[ {Al} \right]background \times \left[ x \right]} \right)} {\left( {\left[ x \right]background \, \times \left[ {Al} \right]} \right)}}} \right. \kern-0pt} {\left( {\left[ x \right]background \, \times \left[ {Al} \right]} \right)}}$$

The element under consideration is represented as ‘*x*’. The background concentrations are the concentrations previously calculated locally. Table 2 provides the EF classes.

**Table 2:** Contamination categories based on EF values (Yongming et al., 2006)

Range	Enrichment factor (EF)
< 2	Deficiency to minimal enrichment
2–5	Moderate enrichment
5–20	Significant enrichment
20–40	Very high enrichment
> 40	Extremely high enrichment

For the EF calculation, the reference concentration should be associated with fine particles, and its concentration should not be anthropogenic (Ackermann, 1980). Typically, elements such as Al, Fe, Mn, and Rb are chosen as references in many studies. Aluminium was chosen as a reference in this study since it is a conservative element and a major constituent of clay minerals, which have been successfully used previously.

By using this type of contamination index, researchers can achieve more precise and inclusive risk evaluation, thereby promoting well-informed decisions regarding environmental preservation and management techniques (Simou et al., 2024).

### Spatial distribution

The GIS mapping explains patterns and regions with elevated PTE concentrations that pose hazards to the environment and human health, as well as identifying the probable causes and contributing factors (Djibril et al., 2026).

Spatial distribution allowed the analysis of elemental distribution patterns in soils, which, combined with multivariate statistics can contribute to understanding the main geochemical elemental relations and infer potential contamination. The interpolation methods used were Ordinary Kriging and Inverse Distance Weighting (IDW) for elements with little difference between their lowest and highest concentrations. All interpolations algorithms were processed using geostatistical analyst menu from software the ArcGIS Pro (v. 3.1.1).

## Results

### Soil PTEs characterisation

The concentrations of Hg, Mo, Cu, Pb, Zn, Ag, Ni, Co, As, Cd, Sb, Cr in the studied soils vary widely: Hg (0.20–20.75 mg kg^−1^), Mo (0.120–1.840 mg kg^−1^), Cu (9.20–60.00 mg kg^−1^), Pb (12.60–449.03 mg kg^−1^), Zn (11.10–95.30 mg kg^−1^), Ag (10–1 164 mg kg^−1^), Ni (4.90–43.00 mg kg^−1^), Co (0.60–16.60 mg kg^−1^), As (14.80–1 431.20 mg kg^−1^), Cd (0.02–0.22 mg kg^−1^), Sb (9.50–>4000.00 mg kg^−1^) and Cr (44.00–113.00 mg kg^−1^) (Table 3).

**Table 3:** Statistical descriptive analysis of PTE contents (mg kg^−1^) in the soils of Ribeiro da Serra

Statistic	Hg	Mo	Cu	Pb	Zn	Ag	Ni	Co	As	Cd	Sb	Cr
Observations	114	54	54	54	54	54	54	54	54	54	54	54
Minimum	0.02	0.12	9.20	12.26	11.10	10.00	4.90	0.60	14.80	0.02	9.50	44.00
Maximum	20.75	1.84	60.00	449.03	95.30	1.16E + 03	43.00	16.60	1.43E + 03	0.22	> 4000	113.00
1st quartile	0.07	1.02	16.05	20.78	22.73	49.25	11.05	1.53	24.53	0.03	36.91	64.00
Median	0.11	1.14	21.95	24.99	26.40	74.50	13.35	2.20	45.55	0.04	80.72	79.50
3rd quartile	0.22	1.35	26.58	33.85	34.58	1.18E + 02	17.68	3.90	106.13	0.07	243.93	87.75
Mean	0.78	1.12	23.09	55.61	30.07	1.60E + 02	14.99	3.24	152.97	0.06	660.29	77.46
Variance	9.47	0.13	119.90	7.40E + 03	222.72	5.93E + 7	41.14	8.33	7.79E + 04	0.00	1.62E + 06	325.42
SD	3.08	0.36	10.95	86.00	14.92	2.43E + 08	6.41	2.89	279.16	0.05	1273.24	18.04
*p*-value *	< 1E − 4	0.0647	< 1E − 4	< 1E − 4	< 1E − 4	< 1E − 4	< 1E − 4	< 1E − 4	< 1E − 4	< 1E − 4	< 1E − 4	0.4169

D, standard deviation; *Shapiro–Wilk normality test

Some elements, as Hg, Pb, Ag, As, and Sb present high variance, mainly controlled by a small number of anomalous samples. For example, Sb exhibits a median concentration of 80.72 mg/kg, while the maximum value exceeds 4000 mg/kg. Similar patterns are observed for As, Hg, Pb, and Ag. These characteristics are consistent with natural geochemical anomalies produced by mineralisation, as the ores from Ribeiro da Serra are enriched in Sb, As, Pb, and contain Ag (electrum) according to the mineral paragenesis identified by Neiva et al. (2008) and Couto (1993). The shown variability is therefore interpreted as reflecting genuine geochemical heterogeneity, as the study area is characterised by mineralised zones with hydrothermal alteration, which commonly produce highly heterogeneous distributions of the main pathfinders related to mineralisation and where the waste piles resultant from mining activities can concentrate them.

The distributions of Pb, Ag, As, and Sb are strongly positively skewed, as shown by the large differences between median and maximum values and by the non-normality tests (*p* < 0.0001), which are typical of geochemical datasets influenced by mineralisation, suggesting the presence of localised geochemical anomalies, where a limited number of samples contain anomalously high concentrations (Reimann et al., 2005).

In the case of Hg, the variability may be enhanced by the occurrence of cinnabar-bearing Carboniferous rocks (Costa et al., 2022) that outcrop northeast of the study area and by the legacy of historical mining and gold processing activities that include Hg amalgams (Carvalho, 1969). The strong positive skewness and the presence of a limited number of highly enriched samples indicate localised geochemical anomalies.

**Fig. 5:**
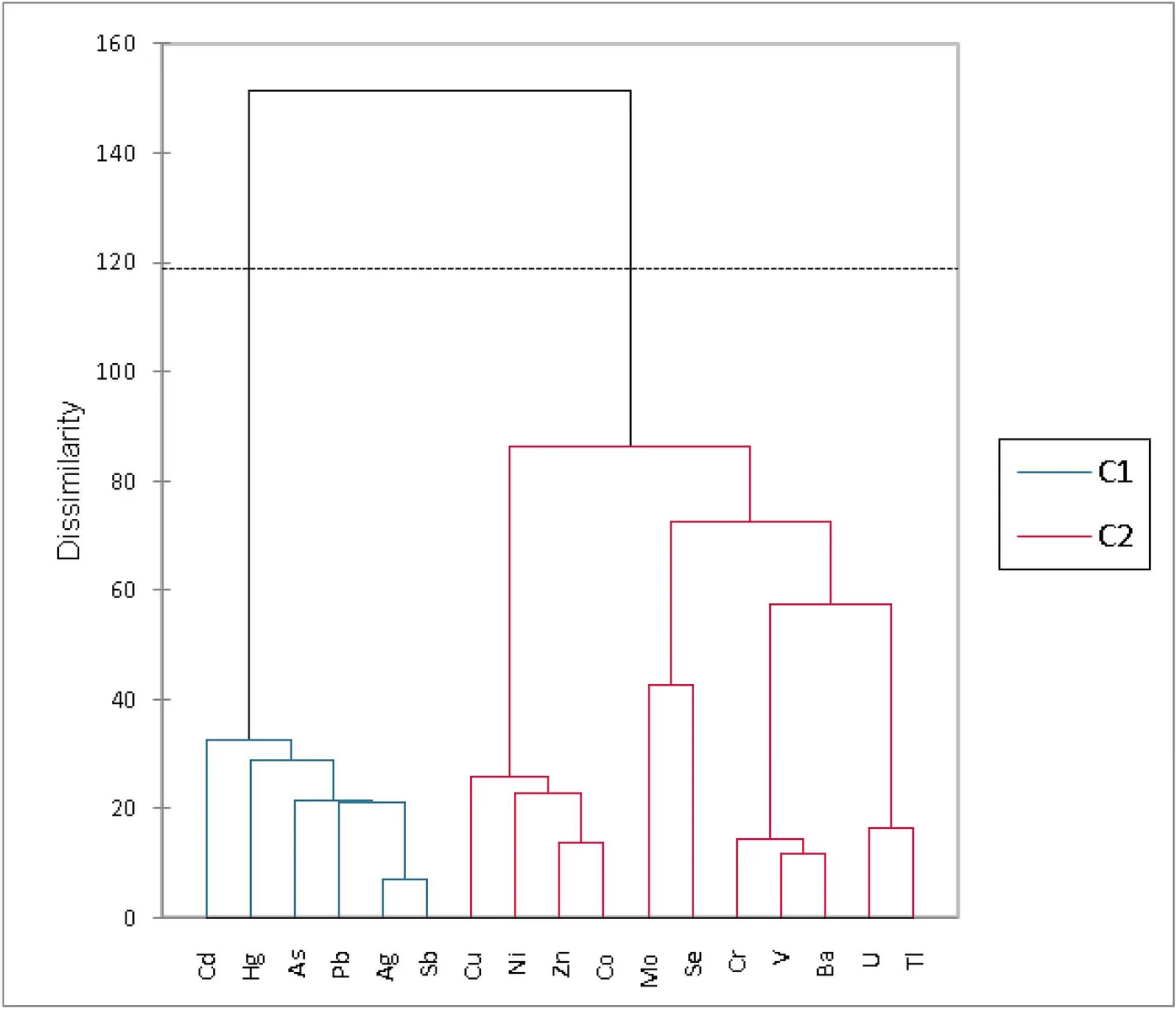
Dendrogram of the PTEs of Ribeiro da Serra

The samples from the area of mining influence (RS037, RS051, RS052, RS066, RS067, RS078, RS112, RS118, RS131, RS143, RS156) (Figure 5) present, overall, the highest contents of PTE.

The Spearman correlation coefficients showed a strong positive correlation (s > 0.7) between Sb–As, Pb–As, Sb–Pb and Ba–V, also highlighting the positive correlation of 0.6 between Hg–Pb and 0.5 between Ag–Hg (Table 4), pointing to a common origin of these elements.

**Table 4:** Spearman correlation for PTEs of Ribeiro da Serra

	Hg	Pb	Zn	Ag	As	Sb	V	Ba	S	Se	Mo	Cd
Hg	1											
Pb	0.6	1										
Zn	0.2	0.2	1									
Ag	0.5	0.6	0.1	1								
As	0.2	0.7	0.0	0.5	1							
Sb	0.2	0.7	0.1	0.5	0.9	1						
V	− 0.6	− 0.5	− 0.4	− 0.6	− 0.3	− 0.3	1					
Ba	− 0.4	− 0.3	− 0.3	− 0.4	− 0.2	− 0.2	0.8	1				
S	0.4	0.4	0.1	0.3	0.4	0.3	− 0.1	0.1	1			
Se	0.0	0.0	0.0	0.0	0.1	0.2	− 0.3	− 0.4	− 0.4	1		
Mo	− 0.6	− 0.7	− 0.1	− 0.7	− 0.7	− 0.7	0.6	0.3	− 0.4	− 0.1	1	
Cd	0.2	0.3	0.3	0.4	0.3	0.4	− 0.2	− 0.1	0.2	− 0.1	− 0.4	1

Similarly, the Cluster analysis (Fig. 5) showed a clear division between the studied PTE. The first cluster is composed of Cd, Hg, As, Pb, Ag, and Sb, and the second cluster is composed of Cu, Ni, Zn, Co, Mn, Se, Cr, V, Ba, U, and Tl.

The first cluster clearly manifests the elemental association representative of mining effects, as mine as the mineralised quartz veins explored in the mine are rich in stibnite (Sb_2_S_3_), berthierite (FeSb_2_S_4_), arsenopyrite (FeAsS), and plagigonite (Pb_5_Sb_8_S_17_) (Neiva et al., 2008).

Adding to the previous, different generations of gold were found in the Sb–Au quartz veins from the Dúrico–Beirão region gold in these veins was associated with arsenopyrite (FeAsS), and electrum (naturally occurring gold-silver alloy) associated with jamesonite (Pb_4_FeSb_6_S_14_) (Couto et al., 1990; Neiva et al., 2008).

Therefore, explored veins mineralogy can explains the presence and association of As, Sb, Pb and Ag, either in pedogenic soils that were developed over mineralised areas or the remnants of the veins that were not processed and were deposited in the waste piles, therefore sharing the same origins. Given that in the ore processing of Au, Hg would be used for amalgamation (alloy) it would form an alloy with both Au and Ag from electrum, explaining the relation shown between Hg and Ag. The resultant ore processing residues should be mainly deposited near the waste piles and processing plant facilities.

**Fig. 6:**
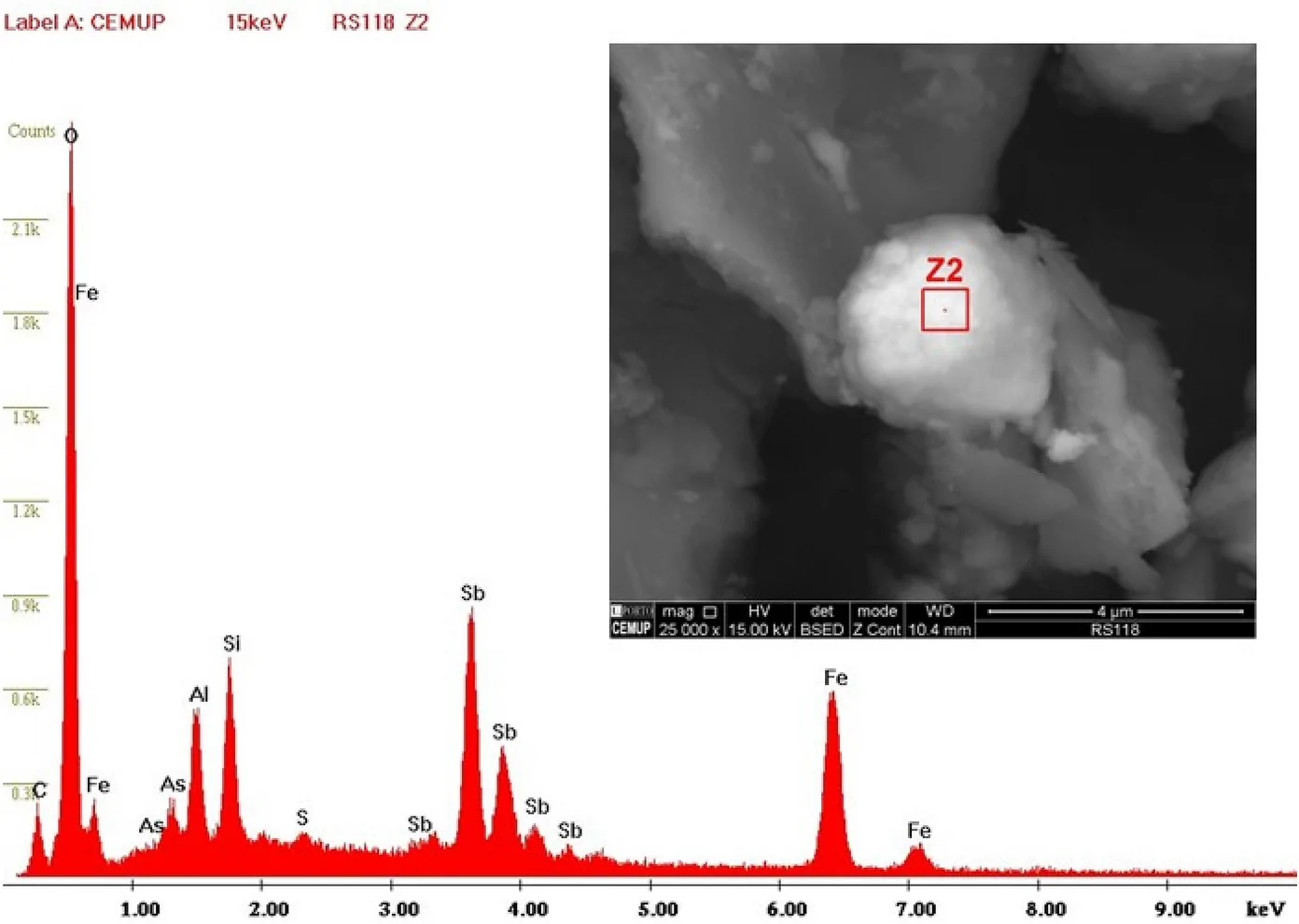
SEM image of soil particle from RS118 and respective EDS spectrum Z2 where it is observed the relationship of Sb with As

The association of Ag with Au mineralisation and the presence of electrum explain why Hg and Ag are in the same cluster, as Ag is known to be associated with Au deposits in Ribeiro da Serra (Reis et al., 2004). Arsenic is an element associated with Sb minerals mining, as arsenopyrite was identified as part of paragenesis, and can be found in soils with As–Sb-rich minerals, as observed from the SEM imaging and EDS spectrum (Fig. 6) of samples from the soils surrounding the mine. The As–Sb association may be related to the leaching and runoff of residues from the waste piles and the pedogenetic enrichment in As and Sb by weathering of mineralised structures.

### Principal component analysis (PCA)

To verify the consistency of geochemical associations an e eventual geochemical controls previously identified by Spearman correlation and cluster analysis, Principal Component Analysis (PCA) was performed.

The number of significant principal components was selected by the Kaiser criterion (Kaiser, 1960). According to the results from PCA (Table 5), four principal components, with eigenvalues that surpassed 1, were extracted cumulatively, accounting for 79% of the total variance in the study area, indicating that they adequately represent the main geochemical patterns within the dataset and most of the geochemical variability within the studied samples.

**Table 5:** Escalated coordinates overview for Ribeiro da Serra study site

Variables	PC1	PC2	PC3	PC4
Hg	**0.69**	0.11	− 0.42	0.36
Pb	**0.85**	− 0.14	0.06	0.07
Zn	0.28	0.25	− **0.51**	− **0.60**
Ag	**0.78**	0.02	− 0.07	0.10
As	**0.74**	− 0.28	0.46	− 0.03
Sb	**0.76**	− 0.25	0.48	− 0.20
V	− **0.74**	− **0.54**	0.21	− 0.08
Ba	− **0.54**	− **0.69**	0.08	− 0.05
S	0.45	− **0.64**	− 0.34	0.26
Se	0.12	**0.67**	**0.57**	0.02
Mo	− **0.89**	0.12	− 0.07	− 0.08
Cd	0.48	− 0.22	− 0.07	− **0.67**
Eigenvalues	5.10	1.91	1.39	1.08
Variance (%)	42.28	15.93	11.54	8.99
Cumulative (%)	42.48	58.41	69.95	78.94

Significant (> 0.5 and <  − 0.5) factor loadings are in bold. The proportion of the total variance captured by a component is given as % Variance and % Cumulative variance at the bottom of the table

The first principal component (PC1) accounts for 42.28% of the total variance. It is characterised by strong positive loadings of Pb (0.85), Ag (0.78), Sb (0.76), As (0.74), and Hg (0.69), and on the other hand, strong negative loadings of Mo (−0.89), V (−0.74), and Ba (−0.54). These results corroborate the elemental relations observed in hierarchical cluster analysis and Spearman correlation. Interpretation suggests that this component represents the dominant mineralisation signature [stibnite (Sb_2_S_3_), berthierite (FeSb_2_S_4_), arsenopyrite (FeAsS), and plagigonite (Pb_5_Sb_8_S_17_), electrum associated with jamesonite (Pb_4_FeSb_6_S_14_) (Couto, 1993; Neiva et al., 2008)], and associated with and the mining residues deposited in the mine waste piles (result of mineral wastes with residual mineralisation and ore processing waste products). The geochemical association between Sb, As is typical of orogenic gold deposits, where these elements commonly occur as pathfinders of gold-bearing fluids. The positive contribution of Pb and Ag is related to the presence of associated sulphide mineralisation rich in Pb and electrum, possibly related to late-stage hydrothermal activity. Nonetheless, the negative loadings of Mo, V, and Ba represent lithological controls and geogenic background geochemical signatures not related to the main ore-forming processes.

The second principal component (PC2) explains 15.93% of the total variance and demonstrates positive loading for Se (0.67) and an association materialised by negative loadings for Ba (−0.69), S (−0.64), and V (−0.54). This component may reflect the presence of lithological units rich in sulphur and vanadium. Given the geological context, the negative Ba–S–V association may be partly related to Carboniferous carbonaceous sequences, including coal-bearing or organic-rich sedimentary layers, which are commonly enriched in V and host disseminated sulphides (as pyrite) and sulphur.

The third principal component (PC3), accounts for 11.54% of the variance of the data, highlighting positive loadings for Se (0.57), Sb (0.48), and As (0.46), and negative loadings for Zn (−0.51) and Hg (−0.42) and the fourth principal component (PC4), explains 8.99% of the variance, and is noted by negative loadings of Cd (−0.67) and Zn (−0.60), opposed to a moderate positive loading of Hg (0.36). These third and fourth components may represent distinct mineralising stages. The positive As–Sb–Se association is consistent with elements of orogenic Sb–Au systems, whereas Zn and Zn–Cd may be associated with a separate sulphide assemblage formed under different mineralising events.

**Fig. 7:**
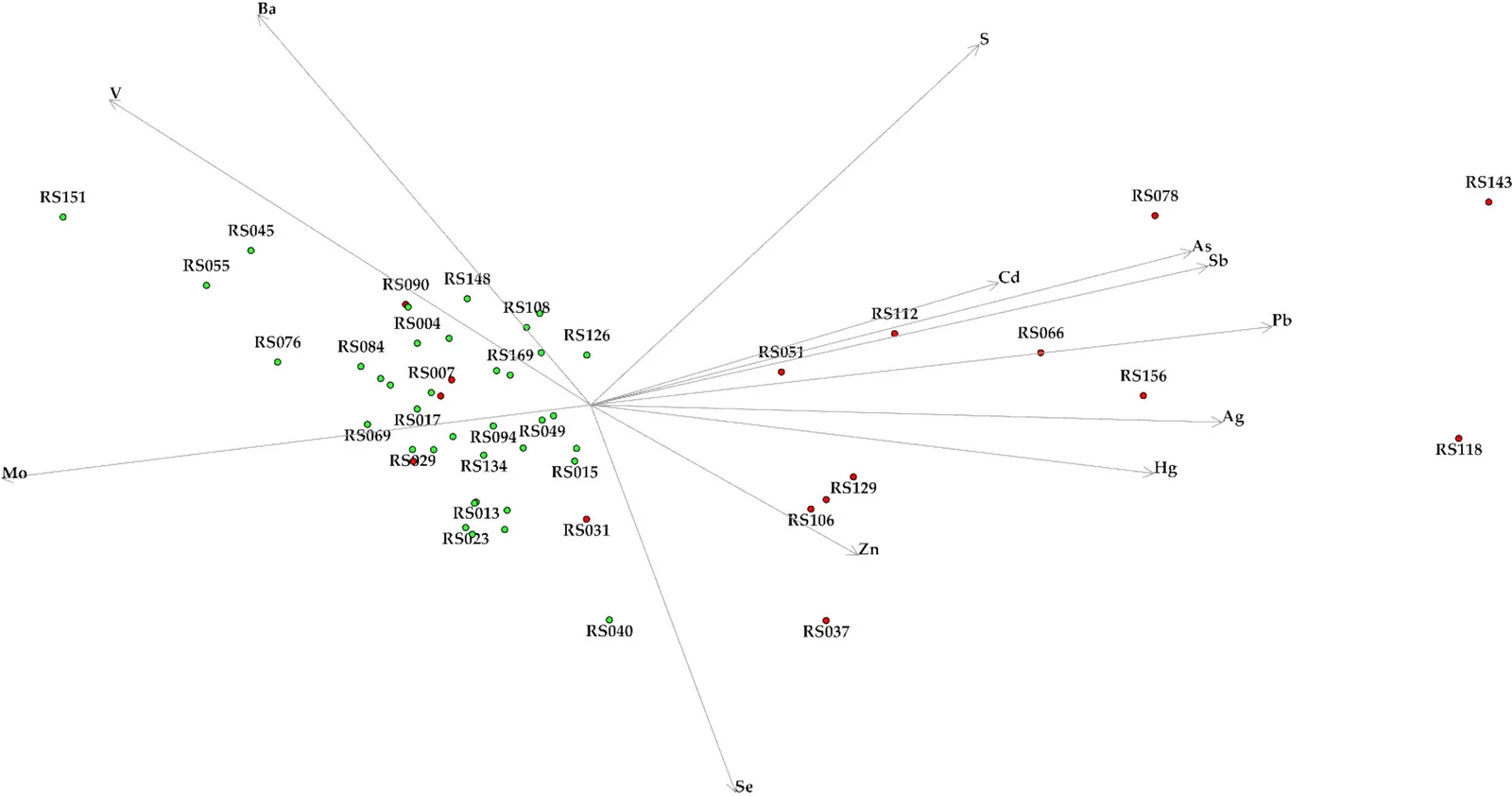
PCA biplot (red, samples from the mining area of influence; green, samples outside the mining area of influence)

The PCA biplot (Fig. 7) shows association pairs between As–Sb, Ag, and Hg associated with Pb. ‘Mine’ samples dominate between the Zn and As vectors. On the other side, a cluster of ‘background’ with punctual ‘mine’ samples is next to the other eigenvectors. A cluster of background associated with Ba, V, Mo, and Se is observed. The cluster of the samples from the mine influence is associated with As, Sb, Pb, Ag, and Hg (Table 5).

Therefore, the PCA supports the interpretation that the high concentration of PTE in the mining influence area could be primarily controlled by mining related processes rather than by the natural substrate alone.

### Soil PTEs enrichment and contamination

The concentration of the PTEs was compared with national reference values for contaminated soils defined by the Portuguese Environmental Agency (APA, 2022) and the Geochemical Atlas (Inácio et al., 2008). The local geochemical background was also determined, considering samples from uphill the mine to account for the effects of natural enrichment due to the local mineralising events. Despite having lower concentration values, for example, regarding Sb and As than soils collected downhill, these local background samples still present an enrichment when compared with the national reference values. Table 6 presents the soil reference values from (APA, 2022), the Geochemical Atlas of Portugal from Inácio et al. (2008), and the local geochemical background. Note that the Geochemical Atlas does not present reference values for Hg and Sb. Figure 9 shows the percentage exceeding different reference value thresholds and, therefore, is considered contaminated.

**Table 6:** Geochemical reference values of PTEs in mg kg^−1^and respective concentrations above threshold

Element	Ref. Val APA (2019)	Conc APA (2019)	Ref. Val Inácio et al. (2008)	Conc Inácio et al. (2008)	Local background	Conc. local
Hg	0.25 (1.8)	8.3x (11.5x)	–	–	0.10	207.5x
Sb	7.5	533.3x	–	–	38.68	103.4x
As	11	130.1	22	65.1x	43.80	32.7x
Ba	390	1.3x	163	1.7x	416.00	1.6x
Cd	1	N/E	–	–	0.04	05.5x
Pb	45	10.0x	34	10.0x	22.85	19.7x
Co	22	N/E	19	N/E	1.70	9.8x
Cu	180	N/E	35	1.7x	18.35	3.3x
Cr	160	N/E	43	N/E	71.50	5.5x
Mo	6.9	N/E	–	–	1.14	1.6x
Ni	130	N/E	43	N/E	12.50	3.4x
Ag	25	N/E	–	–	68,000.00	17.1x
Se	2.4	N/E	–	–	0.15	8.0x
Tl	1	1.2x	–	–	0.53	2.3x
U	23	N/E	–	–	2.65	1.6x
V	86	1.7x	51	2.8x	94.50	1.5x
Zn	340	N/E	85	1.1x	25.00	3.8x

Ref. Val, reference value; Conc, concentrations in comparison with reference value; Conc. Local, concentrations in comparison with local background; N/E, not exceeds; x, times the value exceeds the current reference value

According to APA reference values, Ribeiro da Serra presents high contents within the maximum concentrations of potentially toxic elements of Sb (533x) > As (130x) > Pb (10x) > Hg (8x) (Table 6). Regarding the Geochemical Atlas of Portugal, Ribeiro da Serra presents high maximum concentrations of As (65x) > Pb (10x) > V (3x). Finally, according to the local geochemical background, Ribeiro da Serra presents high maximum concentrations of Hg (208x) > Sb (103x) > As (33x) > Pb (20x).

**Fig. 8:**
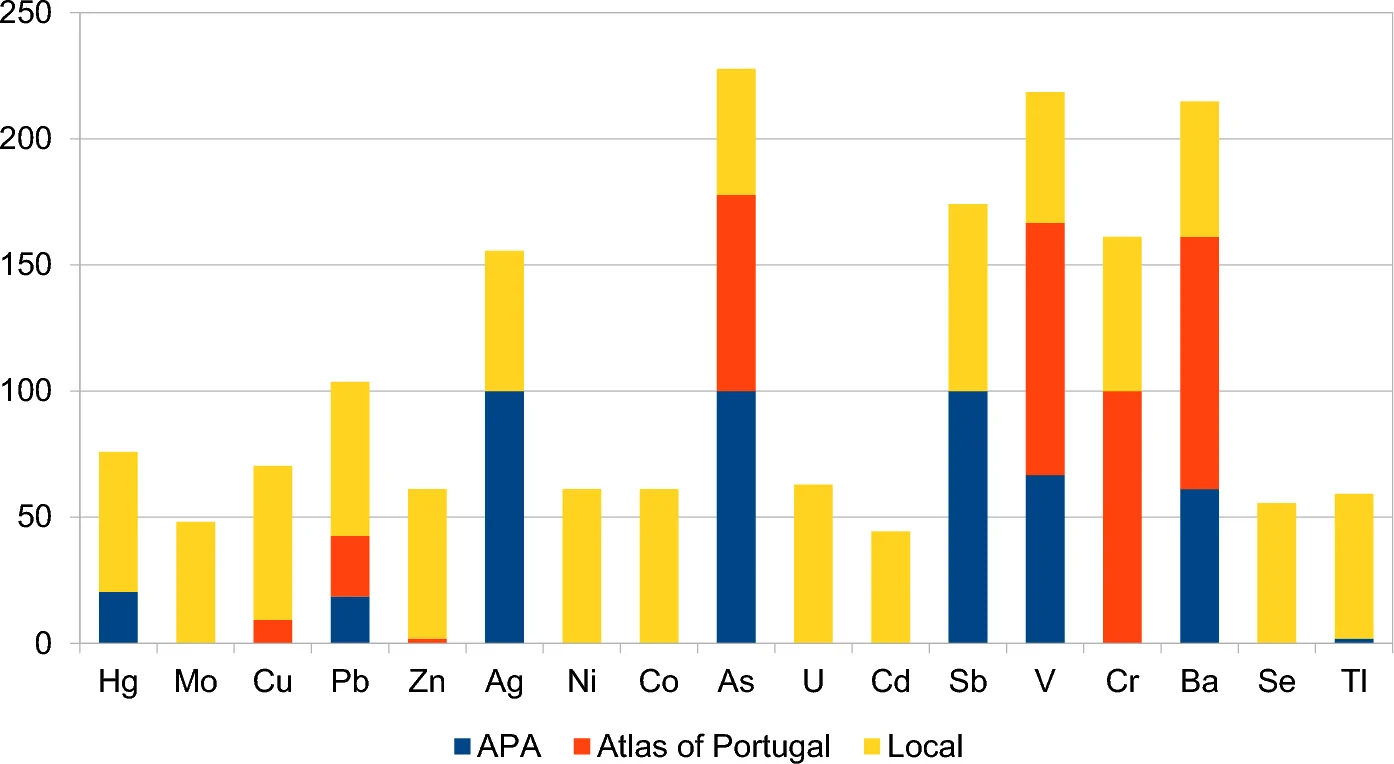
Percentage of contaminated samples according to the reference value for each element source

**Fig. 9:**
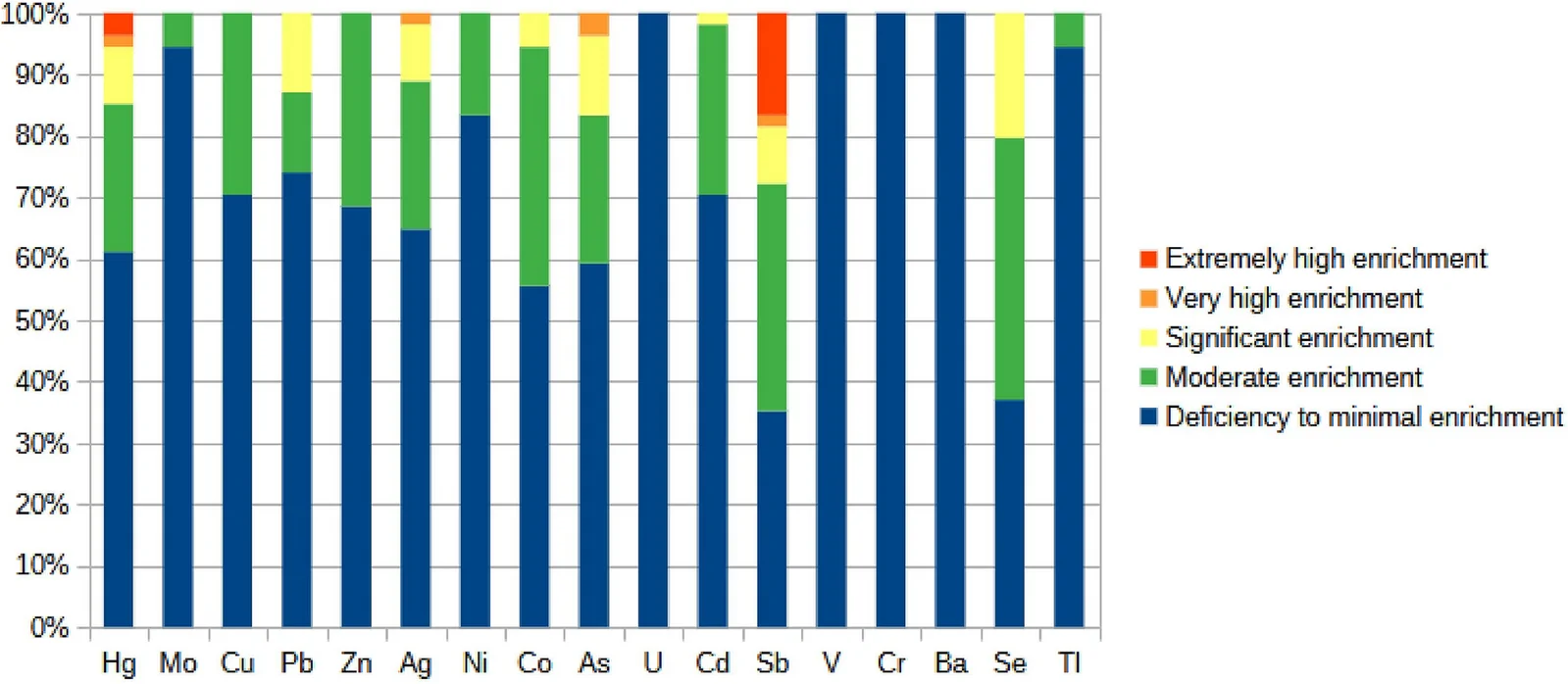
Percentage of samples according to the respective EF class

The average EF observed in the sediments that surround the abandoned mine of Ribeiro da Serra is Hg > Sb > As > Se > Pb > Ag > Co > Cd > Cu > Zn > Ni > Tl > U > Cr > Ba > V > Mo. The key points of the EF analysis are that Hg, Pb, Ag, Co, As, Cd, Sb and Se are the elements that present significant enrichment (Figs. 8, 9). Mercury (EF = 267), antimony (EF = 133) and arsenic (EF = 34) are the only elements with very high to extremely high enrichment. In general, the samples from the drainage valley in the south that actively drain from the mine waste piles present positive anomalies in the enrichment factors, namely, Hg, Pb, Ag, Co, As, Cd, Sb, and Se, posing the greatest ecological risk.

### The As, Sb, Pb and Hg spatial distributions

The spatial distribution modelling was conducted by Geostatistical Analyst from ArcGIS Pro. Regarding As and Pb, a clear spatial structure was identified, allowing the application of ordinary kriging. Different experimental variograms were evaluated for all variables; the hole effect model provided the best cross-validation statistics and was therefore selected for the final interpolation of As, with a nugget of 0.71, sill of 0.81 and range of 199 m. Regarding Pb distribution, the best cross-validation statistics pointed J-Bessel variogram model as the more suitable, with a nugget of 0.35, sill of 0.45 and range of 292 (m). The cross-validation process indicated mean square error (MSE) values close to zero (0.03 in the case of As and 0.00 in the case of Pb), root mean square error (RMSE) values comparable to average standard error (ASE), and Root Mean Square Standardised Error (RMSSE) values close to one, indicate satisfactory model performance.

The experimental variograms of As and Hg presented unsatisfactory variogram model fitting. Consequently, IDW was selected as the interpolation method. The IDW model was implemented using a power value of 2 and a variable search neighbourhood with a maximum of 15 neighbours and a minimum of 10 samples. The search radius was set to 258 m. Cross-validation determined a RMSE of 2.45 mg kg^−1^, regarding Hg, the interpolation results show a low mean error (ME = 80), indicating negligible systematic bias. However, the relatively high RMSE (1134 mg kg^−1^) reflects the strong variability of the dataset and the wide range of values (30–4000 mg kg^−1^), which is typical of heterogeneous geochemical data. Therefore, the model is considered suitable for general trend interpretations but with limited local predictive accuracy.

The interpolations were colour coded from blue (lowest concentration) to red (highest concentration). Blue represents levels under the local reference value (Table 6), while yellow and red mean contamination (determined according to the local reference value) except for the Hg map, in this case it is used APA’s reference value.

The spatial distribution patterns corroborate the relations previously identified by multivariate analysis and indicate different contributions to the source of contamination, primarily originating from the mine waste deposited in the waste piles, as well as from historical industrial ore processing activities.

**Fig. 10:**
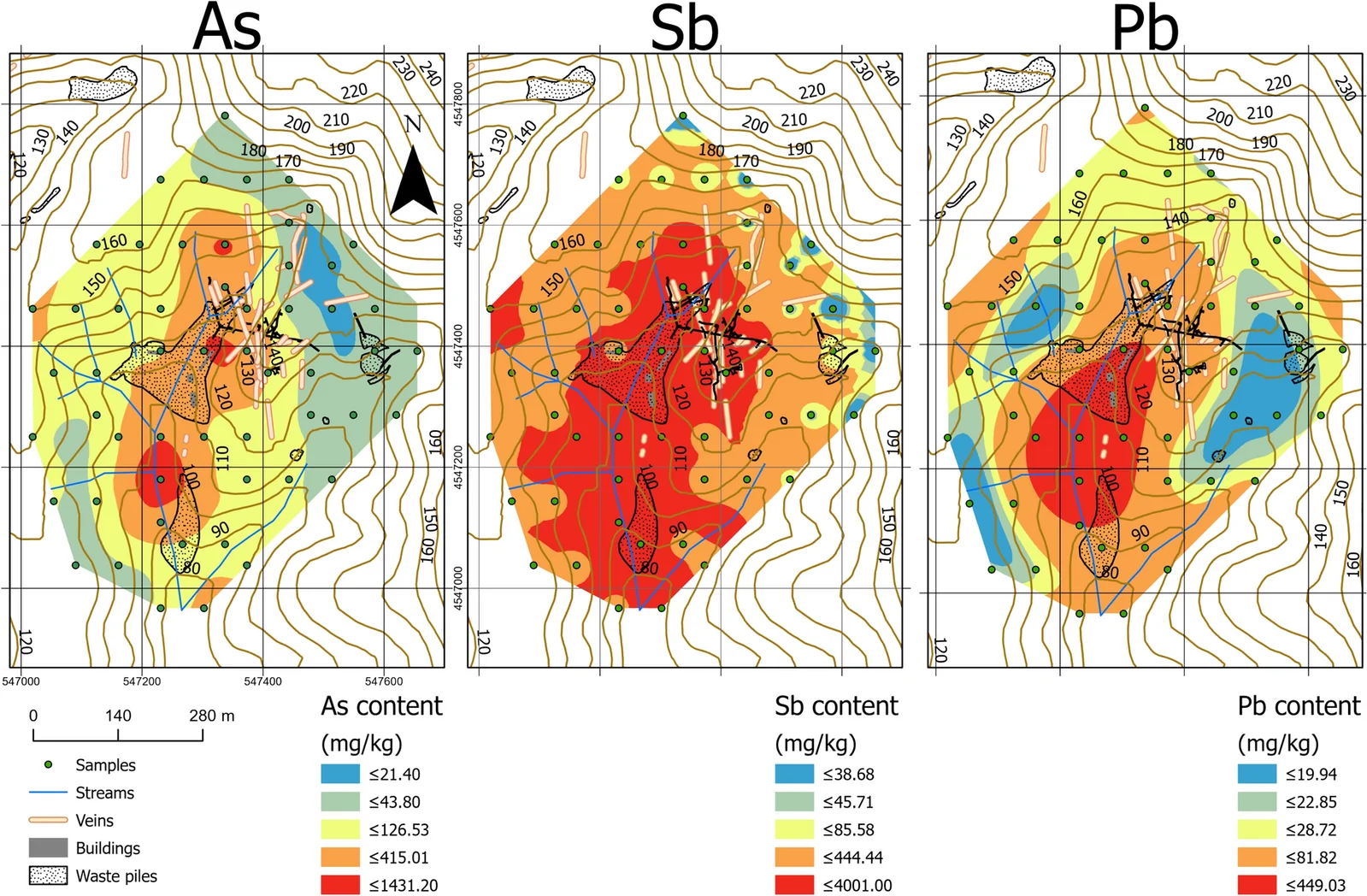
Spatial distribution maps of As, Sb, and Pb contents, coordinates in UTM 29N (WGS 84)

**Fig. 11:**
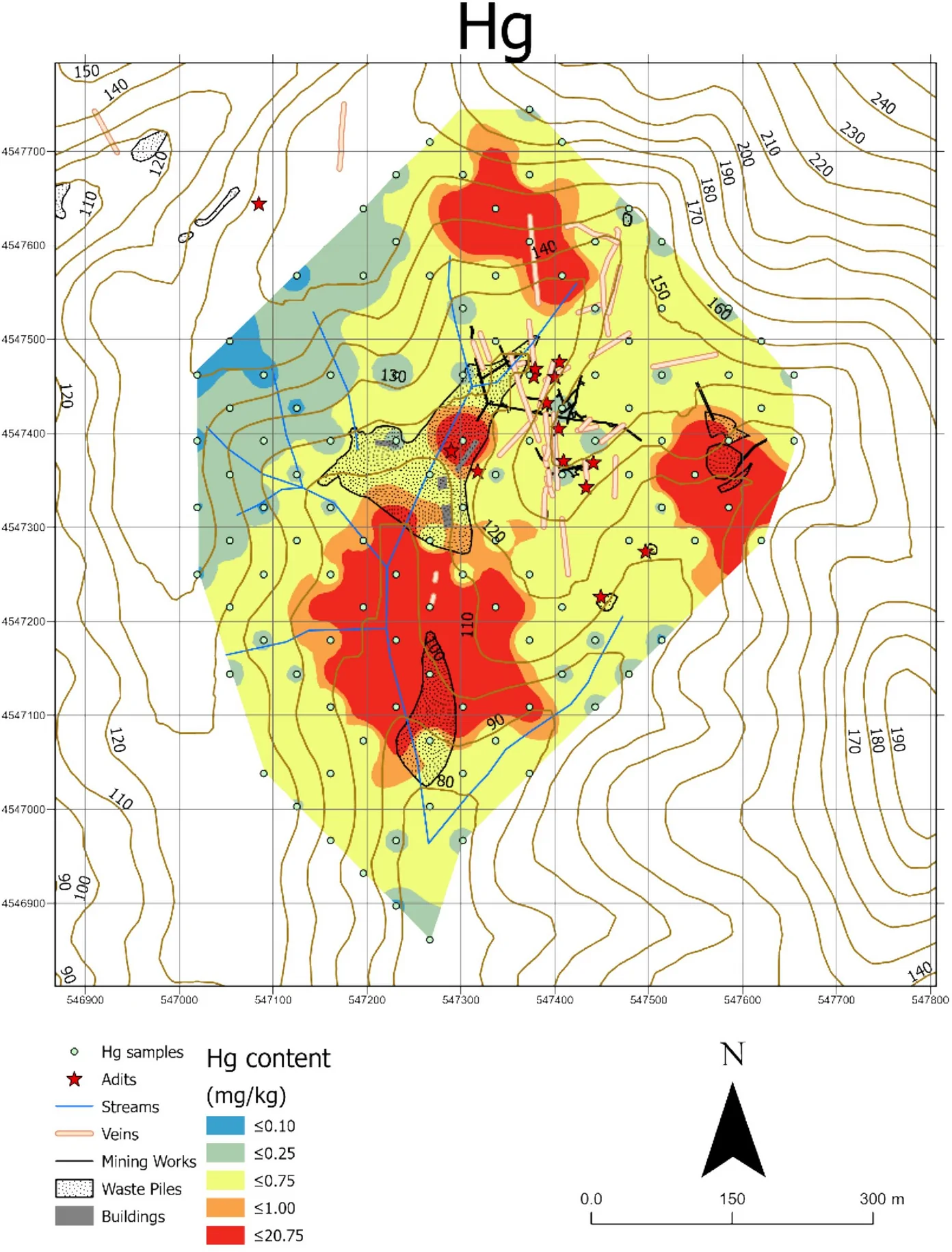
Spatial Distribution maps of Hg in soils, coordinates in UTM 29N (WGS 84)

Figures 10 and 11 show spatial overlap between the highest concentrations of As, Sb, Pb and Hg in the mining drainage area, possibly related to the runoff and leaching from mining residues from the waste piles. There is also a second area enriched located upstream of the mine, in an area with mapped veins that can justify the pedogenetic concentration of As and Sb in soils by weathering of the mineralised structures. The spatial overlap of high concentrations of Hg, As, Sb and Pb is consistent with the associations highlighted in PC1 (Table 6). These elemental concentrations decrease southwards due to dilution and dissipation by the stream runoff, except Sb.

For Ribeiro da Serra and Tapada mines, the study of Carvalho et al. (2024) also demonstrated contamination from the waste piles with a high concentration of PTEs with anomalous values of Sb.

The highest total concentrations of Hg are distributed preferentially in three areas: north and east of the mine, near outcropping carboniferous units (trending NW-SE) and coal mine waste piles, in the mine drainage area, and along the southern waste pile (Fig. 11). These first areas could be associated with a natural pedogenetic enrichment; the area is located uphill from the mine and seems spatially associated with an abandoned coal waste pile. Hg content was evaluated in the different bedrock lithological units (after removal of outliers). The Carboniferous units (0.20 mg kg^−1^) were 60% more enriched in Hg in soils than the Beiras Group (0.08 mg kg^−1^). This natural enrichment can be explained by the Hg association with organic-rich soils, plus the Carboniferous units that outcrop northeast of the study area host coals that were exploited locally and are part of the Douro Carboniferous Basin, where cinnabar was previously identified (Costa et al., 2022).

### Hg mobility in soils

PTEs “total concentration” provides a fair indication of soil contamination in environmental impact assessment studies, however Hg toxicity, bioavailability, and environmental mobility are strongly dependents on its chemical form (Shah et al., 2026). Table 1 presents the speciation and mobility of Hg. Mercury can be retained in soils by adsorption onto organic matter and mineral surfaces affecting its environmental mobility in soil (O’Connor et al., 2019). Once Hg^0^ is released into the soil, it may be oxidised into Hg^2+^. It can immediately form mercuric salts and minerals such as HgCl_2_, HgO, HgS, or, under proper humid conditions, toxic organo-Hg compounds such as CH_3_HgCl (Me-Hg) and (CH_3_)_2_Hg (dimethyl mercury) with biomagnification and bioaccumulation potential in the food chain. The various Hg species in soils have unique properties that affect their fate and transport in soils. Hg_0_ is typically only present in the atmosphere. It can, however, be found as a free phase in soils heavily polluted by anthropogenic activities (e.g., spillages) involving this form of Hg (O’Connor et al., 2019).

Sequential extraction procedures can be applied for metal fractioning and speciation according to species mobility by successively applying extraction solutions with increasing reactivity to the same soil sample and obtaining fractions that contain species with different mobility (from higher to lower mobility) (Monteiro et al., 2024; Mourinha et al., 2022).

**Fig. 12:**
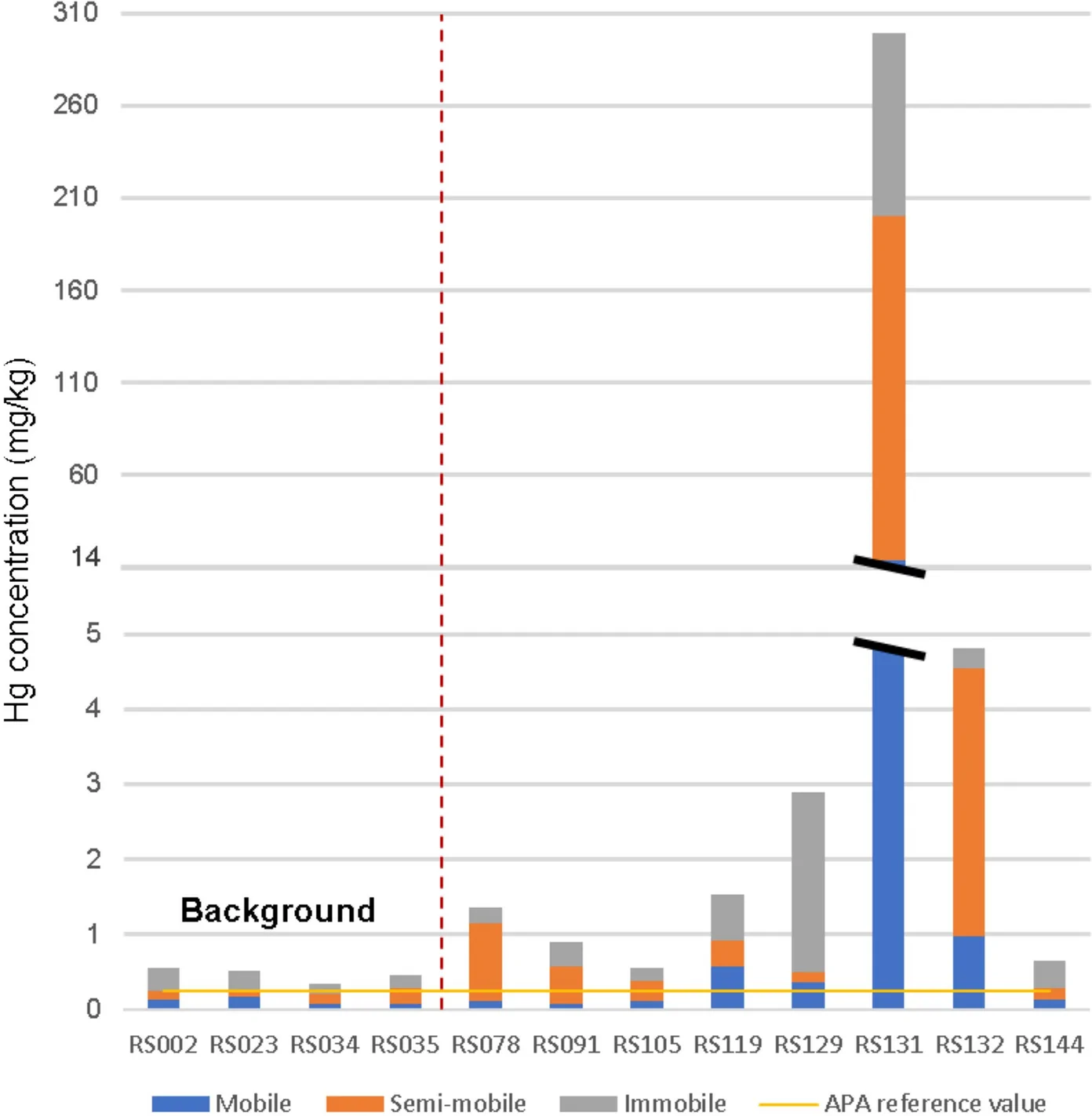
Hg concentrations in the different fractions on background (left) and areas with mine influence (right)

In this study, SEP EPA 3200 (EPA, 2014; Han et al., 2003; Reis et al., 2016) allowed to infer the fractioning and mobility of mercury species in 12 soils samples surrounding the mine, and the results were compiled in Table 7 and Fig. 12. Results confirm that the semi-mobile fraction represented the highest contribution of total mercury content in samples located on the northern waste pile (RS078, RS091, R105), along the drainage of the northern waste pile Sample RS131, and the Southern waste pile (RS132 and RS144).

**Table 7:** Hg contents (in mg kg^−1^) in soil phases in the Ribeiro da Serra samples

Sample	Mobile	Mobile (%)	Semi-mobile	Semi-mobile (%)	Non-mobile	Non-mobile (%)	Total	TOC (%)
RS002	0.14	25.00	0.12	21.43	0.30	53.57	0.56	3.28
RS023	0.17	33.33	0.08	15.69	0.26	50.98	0.51	2.37
RS034	0.08	22.86	0.13	37.14	0.14	40.00	0.35	3.50
RS035	0.06	13.33	0.22	48.89	0.17	37.78	0.45	6.33
RS078	0.11	8.21	1.03	76.87	0.2	14.93	1.34	0.49
RS091	0.06	6.59	0.52	57.14	0.33	36.26	0.91	2.08
RS105	0.10	17.86	0.28	50.00	0.18	32.14	0.56	17.84
RS119	0.57	37.01	0.34	22.08	0.63	40.91	1.54	6.46
RS129	0.35	12.15	0.14	4.86	2.39	82.99	2.88	0.37
RS131	18.72	6.16	186.15	61.23	99.17	32.62	304.04	0.33
RS132	0.97	20.12	3.58	74.27	0.27	5.60	4.82	2.17
RS144	0.13	20.31	0.16	25.00	0.35	54.69	0.64	5.13

Relative precision for mobile, semi-mobile, and non-mobile Hg content was 0.4%, 0.3%, and 0.3%, respectively, obtained by performing assays in duplicate

The background samples (RS002, RS023, RS034) show significantly lower content of total Hg, with a clear predominance in the non-mobile phase (Figs. 11 and 12). Sample RS129 is in a landfill next to former buildings that are not directly related to the mining activities, which can justify the high content of non-mobile Hg. In short, higher non-mobile values are linked to samples upstream of the mine (RS34, RS35) and those from the Carboniferous coal layers (RS02, RS23). These samples exhibit distinct behaviour when compared to samples downstream of the mine.

In general, the soil samples along the waste piles or their drainage areas not only presented higher concentrations of Hg but also presented an enrichment with the most potentially mobile and toxic mercury species, resulting in higher bioavailability potential when compared with Hg levels in areas unaffected by the mine. Samples RS119, RS129, RS131, and RS132 presented mobile Hg concentration levels above the APA reference value (0.25 mg kg^−1^). Samples RS131 and RS132, which are associated with the mine drainage, presented the highest values of semi-mobile and mobile Hg; e.g., sample RS131 has a concerning concentration of mobile Hg, 75 × higher than the APA’s reference value.

The TOC ranges between 0.49 and 17.84% (Table 7), with the highest organic matter contributions in these soils resulting from biomass contributions, as the highest values are in densely vegetated areas. The carboniferous soils potentially enriched with carbon and the soils located upstream the mine, close to the coal contact, also highlight elevated TOC as expected (Fig. 12).

The relationship between Hg and TOC depends heavily on the environment, but in general, TOC influences the retention, transport and bioavailability of mercury, particularly in sediments and natural waters (Chakraborty et al., 2014). In many cases, higher TOC levels mean more organic matter is available to complex with Hg, promoting its sequestration in sediments. Hg tends to bind to organic matter because organic functional groups can adsorb or complex the metal. Consequently, high TOC levels are often associated with higher concentrations of total Hg in the sediment. To evaluate the influence of TOC on the distribution of Hg, a Spearman correlation analysis was conducted (Table 8).

**Table 8:** Spearman correlation between the different Hg fractions and TOC

Hg fraction	Mobile	Semi-mobile	Non-mobile	Total
TOC	0.37	− 0.31	− 0.45	− **0.58**
*p*-values	0.23	0.32	0.14	0.05

**Fig. 13:**
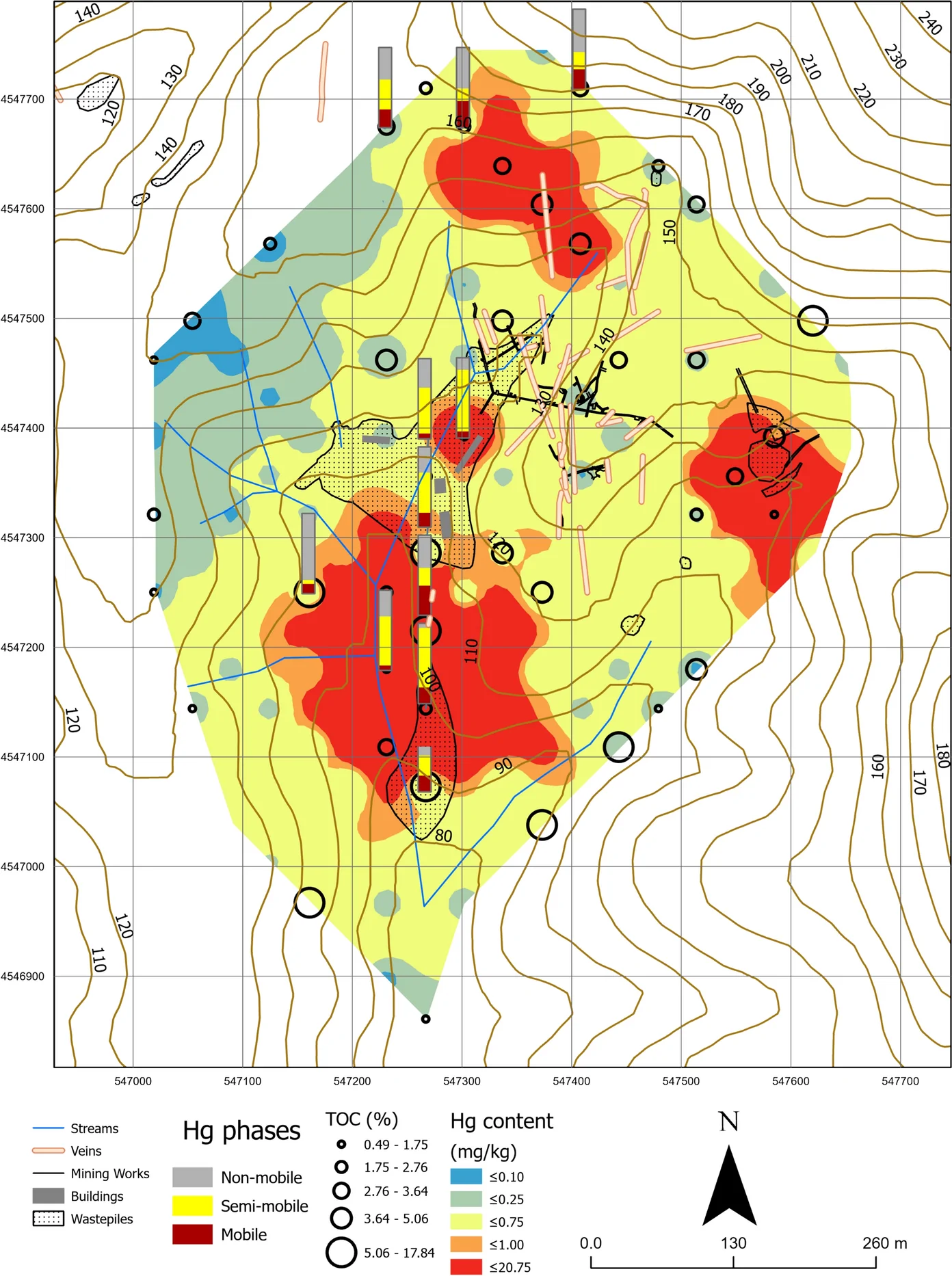
Total Hg spatial distribution map with Hg mobility phases and TOC graduated sample’s location, coordinates in UTM 29N (WGS 84)

Spearman correlation between TOC and the different Hg fractions indicates a statistically significant negative relationship between total Hg concentrations and TOC (rₛ = − 0.58, *p *= 0.046), suggesting higher Hg levels in soils with lower organic carbon content. However, no significant correlations were observed in the mobile, semi-mobile, or non-mobile fractions between TOC and Hg concentrations. Hg distribution appears to be mostly controlled by historical mining contamination and the ore processing activities regardless of the soil organic carbon content. This result is compatible with observations made by Chen et al. (2016) in gold mines in Beijing, where a weak negative correlation was found between the total Hg content TOC. Pestana et al. (2000), also did not find a significant correlation between TOC and Hg concentrations in sediments from Au and Cu mines in Brazil. Xue et al. (2019) studied the anthropogenic influences on Hg in Chinese soils and sediments and its relation with organic carbon and urban areas, concluding that and mining sites shown the largest increase in Hg: TOC ratios, reflecting elevated anthropogenic Hg inputs in these areas, he also highlighted positive correlations between the Hg and TOC concentrations in the background, agricultural, and urban soil but not in the mining soil, where the strong anthropogenic input was the main controller. Figure 13 compares the spatial distributions of total Hg, the different Hg mobility phases, and TOC. The maps did not show evident spatial correlation between TOC and Hg concentration distributions, which is consistent with and further supports the findings previously discussed.

## Conclusions

Despite the mine’s closure two centuries ago, Ribeiro da Serra is still significantly contaminated with high concentrations of PTE such as Hg, As, Sb and Pb, surpassing local geochemical background and APA national reference values.

Enrichment factors confirm very strong contamination, especially for Hg and Sb, and help distinguish the mining-impacted area from natural geochemical background. Overall, the study shows that regional/local geochemical background must be considered when assessing contamination, as natural mineralisation can otherwise be mistaken for pollution.

The spatial distribution of As and Sb shows high concentrations from the north, uphill of the mine, passing through the mine, and following the drainage valley towards the south.

The highest total concentrations of Hg showed a direct association with the mine and its waste piles. These patterns are also supported by the PCA, which shows a strong relation between these elements and the samples from the area influenced by the mining activity.

The highest concentrations of mobile and semi-mobile Hg in the waste piles of the drainage valley are interpreted as the result of gold amalgamation processes. Another site located northeast of the Ribeiro da Serra mine presented high total Hg. Nonetheless, it was found in the waste pile of an abandoned coal concession and, therefore, may be associated with the presence of cinnabar in the coal. Samples between the two Sb–Au waste piles and in the streams’ confluence show concentrations of mobile Hg above the APA’s reference value (0.25 mg kg^−1^), leading to significant environmental risks related to Hg bioaccumulation and soil toxicity.

The Spearman correlation, PCA and AHC effectively pair clusters of elements, offering a strong cross-validation of the detected patterns, improving the understanding of the process affecting the distribution of PTE in the study area.

This study has some limitations to be addressed, the Hg fractionation was performed on only a subset of samples, which may limit how fully the results represent the entire site. In addition, the strong spatial heterogeneity that is typical of abandoned mining areas means that local anomalies may not be fully captured by sampling design. Finally, since the area contains both natural mineralisation and historical mining inputs, distinguishing geochemical background from anthropogenic contamination remains complex.

A highlight of this study is the hypothesis that Hg contamination at the abandoned Sb–Au mine results from the combined effect of historical mining inputs and local geological controls, rather than from mining alone. The study combines mapping, geochemical analysis, and Hg fractionation to show that the highest-risk zones are not only the most contaminated, but also where Hg is most bioavailable and located along the waste pile drainage basins towards population.

Future work should expand Hg fractionation to a larger and more spatially representative sample set, so the patterns observed at Ribeiro da Serra can be better generalised across the study area. It would also be useful to add seasonal monitoring to assess how pluviometry and redox changes affect Hg mobility over time. Further studies should test plant accumulation and groundwater contamination to evaluate the ecological relevance of the mobile Hg, and comparative work with other abandoned Sb–Au sites would help determine whether the geochemical patterns observed here are site-specific or part of a broader behaviour in similar mining environments.

Furthermore, this study offers valuable insights into the ecological and human health risks associated with soil contamination near an abandoned Sb–Au mine that used processing techniques from the 19^th^ century. It can be helpful for informed decision-making by the authorities, emphasising the need for regional geochemical characterisation, especially near mining areas; it shows that the calculation of contamination indexes should not be exclusively based on national or crustal reference values but should include the local (or regional) geochemical background. This approach prevents misidentifying natural mineralisation-induced geochemical anomalies as pollution, ensuring appropriate environmental assessment and management for other former Sb–Au mines. Furthermore, Au is highly sought-after and Sb is a CRM with poor availability. Considering recent technological advancements for extraction and processing in countries like Portugal, Spain and France, this study offers valuable guidance in evaluating the current condition of other abandoned or inactive mines.

## Supplementary Information

Below is the link to the electronic supplementary material.

Supplementary file1 (DOCX 88 KB)

## Data Availability

Data can be provided upon request.

## References

[CR1] Ackermann, F. (1980). A procedure for correcting the grain size effect in heavy metal analyses of estuarine and coastal sediments. *Environmental Technology Letters,**1*(11), 518–527. 10.1080/09593338009384008

[CR2] Afahnwie, N. A., Embui, V. F., Yiika, L. P., et al. (2025). Preliminary stream sediment geochemical exploration for base metals and other elements in terms of source apportionment and contamination status of Manjo and environs, Cameroon. *Discovery Chemistry,**2*, Article 93. 10.1007/s44371-025-00183-2

[CR3] Aitchison, J. (1986). *The statistical analysis of compositional data*. Chapman and. Chapman and Hall.

[CR4] Antunes, I. M. H. R., et al. (2018). Potential toxic elements in stream sediments, soils and waters in an abandoned radium mine (central Portugal). *Environmental Geochemistry and Health,**40*(1), 521–542. 28343275 10.1007/s10653-017-9945-2

[CR5] Antunes, I. M. H. R., Gomes, M. E., Neiva, A. M., Carvalho, P. C., & Santos, A. C. (2016). Potential risk assessment in stream sediments, soils and waters after remediation in an abandoned W > Sn mine (NE Portugal). *Ecotoxicology and Environmental Safety,**133*, 135–145. 27448230 10.1016/j.ecoenv.2016.06.045

[CR6] APA. (2019). *Solos Contaminados–Guia Técnico–Valores de Referência para o Solo*.

[CR7] Barago, N., Pavoni, E., Floreani, F., et al. (2025). Environmental impact and mobility of thallium and other metal(oid)s in soils and tailings near a decommissioned Zn–Pb mine (Raibl, NE Italian Alps). *Environmental Geochemistry and Health,**47*, 89. 10.1007/s10653-025-02400-4 39998704 PMC11861154

[CR8] Barbieri, M. (2016). The importance of enrichment factor (EF) and geoaccumulation index (Igeo) to evaluate the soil contamination. *Journal of Geology & Geophysics*. 10.4172/2381-8719.1000237

[CR9] Barroso, A., Henriques, R., Cerqueira, Â., Gomes, P., Antunes, I. M. H. R., Reis, A. P. M., & Valente, T. M. (2025). Acid mine drainage and waste dispersion in legacy mining sites: An integrated approach using UAV photogrammetry and geospatial analysis. *Journal of Hazardous Materials,**495*, Article 138827. 40482508 10.1016/j.jhazmat.2025.138827

[CR10] Behar, F., Beaumont, V., & De B. Penteado, H. L. (2001). Rock-Eval 6 technology: Performances and developments. *Oil & Gas Science and Technology,**56*(2), 111–134. 10.2516/ogst:2001013

[CR66] Birch, G. F. (2020). Evaluation of sediment contamination indices: A review.*Science of The Total Environment,**723*,138123. 10.1016/j.scitotenv.2020.138123

[CR11] Candeias, C., Ávila, P. F., Ferreira da Silva, E., & Teixeira, J. P. (2015). Integrated approach to assess the environmental impact of mining activities: Estimation of the spatial distribution of soil contamination (Panasqueira mining area, Central Portugal). *Environmental Monitoring and Assessment,**187*(3), 135. 25702148 10.1007/s10661-015-4343-7

[CR12] Carvalho, A. D. de. (1969). Minas de antimónio e ouro de Gondomar. In *Publicações, Estudos, Notas e Trabalhos: Vol. XIX*.

[CR13] Carvalho, M., Cardoso-Fernandes, J., Lima, A., & Teodoro, A. C. (2024). Convolutional neural networks applied to antimony quantification via soil laboratory reflectance spectroscopy in Northern Portugal: Opportunities and challenges. *Remote Sensing,**16*(11), Article 1964. 10.3390/rs16111964

[CR15] Carvalho, P. C. S., Neiva, A. M. R., Silva, M. M. V. G., et al. (2017). Human health risks in an old gold mining area with circum-neutral drainage, Central Portugal. *Environmental Geochemistry and Health,**39*, 43–62. 10.1007/s10653-016-9806-4 26932559

[CR16] Chakraborty, P., Sharma, B., Babu, P. R., Yao, K. M., & Jaychandran, S. (2014). Impact of total organic carbon (in sediments) and dissolved organic carbon (in overlying water column) on Hg sequestration by coastal sediments from the central east coast of India. *Marine Pollution Bulletin,**79*(1–2), 342–347. 24355570 10.1016/j.marpolbul.2013.11.028

[CR17] Chen, X., Ji, H., Yang, W., Zhu, B., & Ding, H. (2016). Speciation and distribution of mercury in soils around gold mines located upstream of Miyun Reservoir, Beijing, China. *Journal of Geochemical Exploration,**163*, 1–9.

[CR18] Costa, M., Moura, H., Pinto de Jesus, A., Suárez-Ruiz, I., & Flores, D. (2022). Effects of magmatic fluids in coals of São Pedro da Cova Coalfield, Douro Carboniferous Basin, Portugal: Insights from inorganic geochemistry. *Minerals,**12*(2), Article 275. 10.3390/min12020275

[CR19] Couto, H. (1993). *As mineralizações de Sb–Au da região Dúrico-Beirã* [Faculdade de Ciências da Universidade do Porto]. http://hdl.handle.net/10216/10678

[CR21] Couto, H., Roger, G., Moëlo, Y., & Bril, H. (1990). Le district à antimoine-or Dúrico-Beirão (Portugal): Évolution paragénétique et géochimique; implications métallogéniques. *Mineralium Deposita,**25*(S1), S69–S81. 10.1007/BF00205252

[CR22] Djibril, K. N. G., Afahnwie, N. A., Yiika, L. P., et al. (2026). Hazardous metal contamination and human-ecological risks in Kouambo-Bipindi surface sediments (Cameroon). *Journal of Sedimentary Environments,**11*, 11. 10.1007/s43217-025-00273-2

[CR23] Durães, N., Portela, L., Sousa, S., Patinha, C., & da Silva, E. F. (2021). Environmental impact assessment in the former mining area of Regoufe (Arouca, Portugal): Contributions to future remediation measures. *International Journal of Environmental Research and Public Health,**18*(3), Article 1180. 33525752 10.3390/ijerph18031180 PMC7908638

[CR24] Eckley, C. S., Gilmour, C. C., Janssen, S., Luxton, T. P., Randall, P. M., Whalin, L., & Austin, C. (2020). The assessment and remediation of mercury contaminated sites: A review of current approaches. *Science of the Total Environment,**707*, Article 136031. 31869604 10.1016/j.scitotenv.2019.136031 PMC6980986

[CR25] EPA. (2014). *Method 3200–Mercury species fractionation and quantification by microwave assisted extraction, selective solvent extraction and/or solid phase extraction*.

[CR26] Espitalie, J., Deroo, G., & Marquis, F. (1985). Rock-eval pyrolysis and its applications (Part two). *Revue De L’institut Français Du Pétrole,**40*, 755–784.

[CR28] Ferreira da Silva, E., et al. (2013). Quantitative–spatial assessment of soil contamination in S. Francisco de Assis due to mining activity of the Panasqueira mine (Portugal). *Environmental Science and Pollution Research,**20*(11), 7534–7549. 23370848 10.1007/s11356-013-1495-2

[CR30] Gomes, P., Valente, T., & Font, E. (2025). Integrated characterization of sediments contaminated by acid mine drainage: Mineralogical, magnetic, and geochemical properties. *Minerals,**15*(8), 786.

[CR32] Han, Y., Kingston, H. M., Boylan, H. M., Rahman, G. M. M., Shah, S., Richter, R. C., Link, D. D., & Bhandari, S. (2003). Speciation of mercury in soil and sediment by selective solvent and acid extraction. *Analytical and Bioanalytical Chemistry,**375*(3), 428–436. 10.1007/s00216-002-1701-4 12589509

[CR65] Hou, D., O'Connor, D., Nathanail, P., Tian, L., & Ma, Y. (2017). Integrated GIS and multivariate statistical analysis forregional scale assessment of heavy metal soil contamination: A critical review. *Environmental Pollution,**231*(Part 1),1188–1200. 10.1016/j.envpol.2017.07.021 28939126

[CR33] Inácio, M., Pereira, V., & Pinto, M. (2008). The soil geochemical atlas of Portugal: Overview and applications. *Journal of Geochemical Exploration,**98*(1–2), 22–33. 10.1016/j.gexplo.2007.10.004

[CR34] Kabata-Pendias, A., & Mukherjee, A. B. (2007). *Trace elements from soil to human*.

[CR35] Kaiser, H. F. (1960). The application of electronic computers to factor analysis. *Educational and Psychological Measurement,**20*(1), 141–151. 10.1177/001316446002000116

[CR36] Kernow Mining Portugal. (2008). *Sobrido–3° Relatório de Actividades*.

[CR37] Kernow Mining Portugal. (2009). *Sobrido–5° Relatório de Actividades*.

[CR38] Konanç, M. U., Değermenci, G. D., Kariper, İA., & Yavuz, E. (2024). After-effects of a closed copper mine: Detailed analysis of environmental impacts in soil and plant samples. *Environmental Earth Sciences,**83*, Article 412. 10.1007/s12665-024-11725-9

[CR39] Monteiro, M., Santos, P., Marques, J. E., et al. (2024). Assessment of mobile mercury concentration in soils of an abandoned coalfield waste pile in Douro region: The Fojo waste pile (Portugal) study case. *Journal of Soils and Sediments,**24*, 2068–2077. 10.1007/s11368-024-03786-x

[CR68] Monteiro, M., Santos, P., Espinha Marques, J., Flores, D., Azenha, M., & Ribeiro, J. A. (2025). Assessment of PotentialEnvironmental Risks Posed by Soils of a Deactivated Coal Mining Area in Northern Portugal—Impact of Arsenic and Antimony. *Pollutants,**5*(2), 15. 10.3390/pollutants5020015

[CR40] Mourinha, C., et al. (2022). Potentially toxic elements’ contamination of soils affected by mining activities in the Portuguese sector of the Iberian Pyrite Belt and optional remediation actions: A review. *Environments,**9*(1), Article 11.

[CR41] Moyo, A., Parbhakar-Fox, A., Meffre, S., & Cooke, D. (2023). Geoenvironmental characterisation of legacy mine wastes from Tasmania—Environmental risks and opportunities for remediation and value recovery. *Journal of Hazardous Materials,**454*, Article 31521. 10.1016/j.jhazmat.2023.131521 37146342

[CR42] Neiva, A. M. R., Andráš, P., & Ramos, J. M. F. (2008). Antimony quartz and antimony–gold quartz veins from northern Portugal. *Ore Geology Reviews,**34*(4), 533–546. 10.1016/j.oregeorev.2008.03.004

[CR43] O’Connor, D., Hou, D., Ok, Y. S., Mulder, J., Duan, L., Wu, Q., Wang, S., Tack, F. M. G., & Rinklebe, J. (2019). Mercury speciation, transformation, and transportation in soils, atmospheric flux, and implications for risk management: A critical review. *Environment International,**126*, 747–761. 10.1016/j.envint.2019.03.019 30878870

[CR44] Paruchuri, Y., Siuniak, A., Johnson, N., Levin, E., Mitchell, K., Goodrich, J. M., Renne, E. P., & Basu, N. (2010). Occupational and environmental mercury exposure among small-scale gold miners in the Talensi-Nabdam District of Ghana’s Upper East region. *Science of the Total Environment,**408*(24), 6079–6085. 20875913 10.1016/j.scitotenv.2010.08.022 PMC4083620

[CR45] Pestana, M. H. D., Lechler, P., Formoso, M. L. L., & Miller, J. (2000). Mercury in sediments from gold and copper exploitation areas in the Camaqua River Basin, Southern Brazil. *Journal of South American Earth Sciences,**13*(6), 537–547.

[CR46] Reimann, C., Filzmoser, P., & Garrett, R. G. (2005). Background and threshold: Critical comparison of methods of determination. *Science of the Total Environment,**346*(1–3), 1–16. 15993678 10.1016/j.scitotenv.2004.11.023

[CR47] Reis, A. T., Davidson, C. M., Vale, C., & Pereira, E. (2016). Overview and challenges of mercury fractionation and speciation in soils. In *TrAC–Trends in analytical chemistry* (Vol. 82, pp. 109–117). Elsevier. 10.1016/j.trac.2016.05.008

[CR48] Reis, A. T., Rodrigues, S. M., Davidson, C. M., Pereira, E., & Duarte, A. C. (2010). Extractability and mobility of mercury from agricultural soils surrounding industrial and mining contaminated areas. *Chemosphere,**81*(11), 1369–1377. 20932549 10.1016/j.chemosphere.2010.09.030

[CR49] Reis, A. P., Sousa, A. J., Ferreira da Silva, E., Patinha, C., & Fonseca, E. C. (2004). Combining multiple correspondence analysis with factorial kriging analysis for geochemical mapping of the gold–silver deposit at Marrancos (Portugal). *Applied Geochemistry,**19*(4), 623–631. 10.1016/j.apgeochem.2003.09.003

[CR51] Sant’Ovaia, H., Cruz, C., Guedes, A., Ribeiro, H., Santos, P., Pereira, S., Espinha Marques, J., Ribeiro, M. D., Mansilha, C., Martins, H. C. B., Valentim, B., Torres, J., Abreu, I., Noronha, F., & Flores, D. (2023). Contamination fingerprints in an inactive W (Sn) mine: The regoufe mine study case (Northern Portugal). *Minerals,**13*(4), Article 497. 10.3390/min13040497

[CR52] Santos, P., Ribeiro, J., Espinha Marques, J., & Flores, D. (2023). Environmental and health risk assessment of soil adjacent to a self-burning waste pile from an abandoned coal mine in northern Portugal. *Environments,**10*(3), 53. 10.3390/environments10030053

[CR54] Shah, N., Awais, M., Ali, M., & Li, H. (2026). Speciation-driven toxicity and remediation of mercury: Mechanistic insights and policy implications. *Journal of Hazardous Materials Advances*. 10.1016/j.hazadv.2026.101233

[CR55] Shi, B., Meng, J., Wang, T., Li, Q., Zhang, Q., & Su, G. (2024). The main strategies for soil pollution apportionment: A review of the numerical methods. *Journal of Environmental Sciences,**136*, 95–109. 10.1016/j.jes.2022.09.027 37923480

[CR56] Sigué, C., Yewong, N. E., Yiika, L. P., et al. (2025). Assessment of contamination, sources and health risks of potentially hazardous elements in surface sediments of Dibang, Cameroon. *Discovery Environment,**3*, Article 165. 10.1007/s44274-025-00363-y

[CR67] Simou, A., Sarti, O., Abdelfattah, B., Mrabet, A., Khaddor, M., & Allali, N. (2024). Assessing ecological and health risksof potentially toxic elements in marine and beach sediments of Tangier Bay, Southwestern Mediterranean sea.*Marine Pollution Bulletin,**209*(Pt B), 117234. 10.1016/j.marpolbul.2024.117234 39522119

[CR57] Soetan, O., Viteritto, M., Qian, Y., & Feng, H. (2024). Evaluation of toxic metal pollution in freshwater surficial sediments using environmental indices and multivariate statistical approaches—A systematic review. *Environmental Nanotechnology, Monitoring & Management,**22*, Article 100961.

[CR58] Swain, C. K. (2024). Environmental pollution indices: A review on concentration of heavy metals in air, water, and soil near industrialization and urbanisation. *Discover Environment,**2*(1), 5.

[CR59] Telmer, K.H., Stapper, D., 2007. Evaluating and monitoring small scale mining and mercury use: Building a knowledge base with satellite imagery and field work. UNDP/GEF/UNIDO Project EG/GLO/01/G34. Final report to the United Nations Industrial Development Organisation, Vienna.

[CR60] UNEP. (2012). *Reducing Mercury Use in Artisanal and Small-scale Gold Mining - A Practical Guide*. www.artisanalgoldcouncil.org

[CR61] Valente, T. M., & Gomes, C. L. (2009). Occurrence, properties and pollution potential of environmental minerals in acid mine drainage. *Science of the Total Environment,**407*(3), 1135–1152. 19004477 10.1016/j.scitotenv.2008.09.050

[CR69] Xue, W., Kwon, S., Li, Y., Zhang, Y., Liu, M., Wang, J., … & Yin, R. (2019). Anthropogenic infl uences on mercury inChinese soil and sediment revealed by relationships with total organic carbon.*Environmental Pollution,**255*(Part 1),113190. 10.1016/j.envpol.2019.113186 31520907

[CR63] Yiika, L. P., Tita, M. A., Suh, C. S., Mimba, M. E., & Ndema Mbongué, J. L. (2023). Heavy metal speciation by Tessier sequential extraction applied to artisanal gold mine tailings in Eastern Cameroon. *Chemistry Africa,**6*, 2705–2723. 10.1007/s42250-023-00652-0

[CR64] Yongming, H., Peixuan, D., Junji, C., & Posmentier, E. S. (2006). Multivariate analysis of heavy metal contamination in urban dusts of Xi’an, Central China. *Science of the Total Environment,**355*(1–3), 176–186. 10.1016/j.scitotenv.2005.02.026 15885748

