# Supplementary material for: Soil contamination assessment of a 19th-century abandoned Sb–Au mine in northern Portugal
Source: Environ Geochem Health. 2026 Jul 20;48(11):472. doi: 10.1007/s10653-026-03359-6 (PMC13385249; doi:10.1007/s10653-026-03359-6)
Supplement: Supplementary file 1 — Supplementary file1 (DOCX 88 KB) [file 10653_2026_3359_MOESM1_ESM.docx]

**Appendix 1. Inductively Coupled Plasma Mass Spectrometry (ICP-MS***)*

ICP-MS was conducted in Bureau Veritas Laboratory, in Vancouver, Canada. The analytical dataset demonstrates high precision and generally good accuracy, based on standard QA/QC assessment. Pulp duplicate samples show excellent reproducibility, with most relative percent differences (RPD) below 5% and only minor deviations approaching 10%, indicating low analytical variability and good laboratory consistency. Certified reference materials (OREAS standards) display consistent results between replicates and broadly align with expected values, suggesting acceptable analytical accuracy with no significant systematic bias, although slight variability is observed for certain elements such as Ag and Sb. Blank samples indicate negligible contamination.

Table S1 - ICP-MS geochemical data from Ribeiro da Serra soils.

| **Samples** | **Mo** | **Cu** | **Pb** | **Zn** | **Ag** | **Ni** | **Co** | **As** | **Cd** | **Sb** | **V** | **Cr** | **Ba** | **Ti** | **Se** |
| --- | --- | --- | --- | --- | --- | --- | --- | --- | --- | --- | --- | --- | --- | --- | --- |
| SJ6 | 1.87 | 19.0 | 27.28 | 46.0 | 113 | 20.2 | 19.3 | 20.5 | 0.07 | 2.34 | 89 | 61 | 363 | 0.263 | <0.3 |
| SJ7 | 3.19 | 21.2 | 28.25 | 32.9 | 102 | 20.8 | 5.5 | 33.6 | 0.04 | 2.56 | 138 | 76 | 456 | 0.301 | 0.8 |
| SJ8 | 1.98 | 18.8 | 26.85 | 44.2 | 103 | 20.7 | 18.1 | 21.5 | 0.06 | 2.20 | 97 | 64 | 380 | 0.282 | 0.4 |
| SJ9 | 1.83 | 19.0 | 25.33 | 52.8 | 112 | 22.2 | 21.0 | 20.6 | 0.06 | 1.97 | 84 | 61 | 348 | 0.270 | 0.4 |
| SJ10 | 4.47 | 28.9 | 35.01 | 35.5 | 78 | 22.3 | 13.9 | 27.9 | 0.06 | 3.62 | 129 | 84 | 437 | 0.322 | 0.7 |
| SJ11 | 1.67 | 18.5 | 24.68 | 44.2 | 108 | 19.6 | 13.3 | 26.6 | 0.04 | 2.01 | 87 | 59 | 344 | 0.254 | 0.4 |
| SJ12 | 1.51 | 13.4 | 20.24 | 31.5 | 90 | 16.3 | 11.0 | 14.7 | 0.05 | 1.95 | 65 | 51 | 266 | 0.227 | <0.3 |
| SJ13 | 1.88 | 15.7 | 24.56 | 36.5 | 91 | 24.1 | 9.2 | 17.0 | 0.03 | 2.17 | 79 | 58 | 344 | 0.274 | 0.3 |
| SJ14 | 2.56 | 20.8 | 27.27 | 45.0 | 129 | 27.4 | 23.4 | 20.6 | 0.04 | 2.61 | 95 | 68 | 366 | 0.286 | 0.5 |
| RS001 | 1.01 | 9.5 | 33.49 | 12.7 | 72 | 10.2 | 1.5 | 25.0 | 0.02 | 13.38 | 99 | 47 | 459 | 0.365 | <0.3 |
| RS004 | 0.70 | 9.2 | 17.05 | 25.1 | 35 | 10.3 | 3.1 | 24.5 | 0.03 | 12.79 | 60 | 47 | 437 | 0.126 | <0.3 |
| RS007 | 1.19 | 16.2 | 22.23 | 24.5 | 91 | 13.7 | 5.1 | 18.7 | 0.04 | 10.71 | 77 | 60 | 523 | 0.207 | <0.3 |
| RS013 | 1.83 | 13.9 | 22.19 | 22.7 | 120 | 12.5 | 3.9 | 24.6 | 0.04 | 23.40 | 80 | 61 | 352 | 0.211 | 0.3 |
| RS015 | 1.17 | 13.9 | 28.89 | 49.1 | 98 | 17.6 | 3.9 | 16.0 | 0.07 | 14.55 | 71 | 56 | 407 | 0.231 | <0.3 |
| RS017 | 1.32 | 16.0 | 23.97 | 23.7 | 114 | 13.3 | 5.1 | 17.4 | 0.03 | 9.95 | 80 | 60 | 479 | 0.221 | <0.3 |
| RS019 | 1.35 | 12.7 | 21.08 | 14.7 | 88 | 9.4 | 1.4 | 21.3 | 0.03 | 9.50 | 79 | 60 | 390 | 0.225 | <0.3 |
| RS021 | 1.78 | 17.1 | 28.76 | 36.5 | 101 | 15.2 | 2.8 | 22.6 | 0.05 | 33.94 | 98 | 65 | 351 | 0.229 | 0.4 |
| RS023 | 1.42 | 20.1 | 26.20 | 41.1 | 53 | 17.8 | 4.0 | 21.0 | 0.05 | 30.32 | 108 | 73 | 381 | 0.253 | 0.5 |
| RS027 | 1.72 | 11.0 | 24.53 | 31.1 | 124 | 14.3 | 3.3 | 17.9 | 0.05 | 16.87 | 86 | 67 | 374 | 0.246 | 0.4 |
| RS029 | 1.56 | 9.2 | 20.64 | 29.1 | 70 | 11.3 | 1.8 | 14.8 | 0.04 | 9.54 | 85 | 55 | 268 | 0.216 | <0.3 |
| RS031 | 1.20 | 17.5 | 20.93 | 29.8 | 67 | 12.7 | 2.3 | 20.3 | 0.07 | 25.73 | 88 | 64 | 296 | 0.371 | 0.4 |
| RS034 | 1.05 | 17.7 | 19.54 | 24.5 | 54 | 11.2 | 1.7 | 112.9 | 0.03 | 47.03 | 98 | 86 | 436 | 0.379 | 0.9 |
| RS037 | 1.38 | 19.0 | 47.29 | 49.8 | 110 | 19.5 | 2.5 | 1431.2 | <0.02 | 575.47 | 90 | 44 | 269 | 0.674 | 1.1 |
| RS040 | 1.43 | 20.9 | 33.97 | 50.9 | 98 | 43.0 | 11.6 | 28.6 | 0.08 | 59.42 | 114 | 72 | 400 | 1.137 | 0.6 |
| RS045 | 1.14 | 31.2 | 17.06 | 15.0 | 92 | 23.6 | 1.0 | 47.8 | 0.03 | 19.46 | 142 | 93 | 664 | 0.382 | <0.3 |
| RS049 | 1.06 | 22.4 | 24.82 | 28.3 | 44 | 14.7 | 1.6 | 111.0 | 0.06 | 162.61 | 100 | 85 | 426 | 0.319 | 0.4 |
| RS051 | 0.78 | 23.7 | 31.82 | 26.1 | 68 | 13.0 | 2.3 | 225.0 | 0.22 | 2653.16 | 104 | 100 | 487 | 0.255 | 0.3 |
| RS052 | 1.19 | 51.5 | 40.35 | 38.6 | 308 | 25.1 | 6.3 | 120.9 | 0.11 | 2214.49 | 97 | 80 | 458 | 0.403 | 0.7 |
| RS055 | 0.76 | 21.7 | 12.64 | 23.6 | <20 | 18.7 | 1.4 | 21.2 | 0.03 | 50.68 | 112 | 76 | 522 | 0.403 | <0.3 |
| RS057 | 1.18 | 28.0 | 20.98 | 39.7 | 173 | 22.4 | 6.1 | 20.8 | 0.04 | 30.04 | 97 | 84 | 425 | 0.293 | 0.5 |
| RS061 | 1.04 | 15.7 | 25.48 | 25.9 | 49 | 11.0 | 1.1 | 30.8 | 0.10 | 82.11 | 82 | 64 | 337 | 0.327 | <0.3 |
| RS066 | 0.60 | 27.8 | 197.01 | 17.5 | 771 | 14.0 | 3.2 | 501.9 | 0.19 | >4000.00 | 117 | 108 | 530 | 0.333 | 0.4 |
| RS067 | 1.14 | 22.2 | 28.50 | 21.0 | 23 | 22.2 | 1.6 | 129.6 | 0.03 | 1103.06 | 117 | 95 | 476 | 0.344 | 0.5 |
| RS069 | 1.14 | 26.8 | 23.63 | 26.3 | 36 | 25.9 | 4.0 | 29.6 | 0.04 | 170.90 | 129 | 110 | 549 | 0.328 | 0.8 |
| RS073 | 1.10 | 25.1 | 34.84 | 36.1 | 66 | 13.0 | 1.8 | 84.8 | 0.05 | 342.44 | 102 | 86 | 469 | 0.310 | <0.3 |
| RS076 | 1.84 | 10.9 | 20.77 | 14.0 | <20 | 4.9 | 0.8 | 85.2 | 0.03 | 59.27 | 93 | 81 | 380 | 0.209 | 0.7 |
| RS078 | 1.16 | 25.9 | 171.89 | 51.8 | 502 | 13.2 | 3.8 | 680.5 | 0.06 | >4000.00 | 143 | 113 | 652 | 0.358 | <0.3 |
| RS084 | 1.13 | 12.4 | 17.59 | 22.3 | 49 | 8.5 | 1.4 | 21.1 | 0.03 | 59.11 | 91 | 79 | 396 | 0.239 | <0.3 |
| RS088 | 1.19 | 24.4 | 20.79 | 22.8 | 29 | 10.2 | 1.8 | 34.8 | 0.04 | 89.74 | 90 | 76 | 357 | 0.162 | 1.2 |
| RS090 | 1.10 | 22.8 | 22.39 | 25.0 | 37 | 15.5 | 1.7 | 76.7 | 0.06 | 101.96 | 120 | 88 | 520 | 0.314 | <0.3 |
| RS094 | 1.13 | 23.8 | 24.93 | 26.5 | 54 | 12.7 | 1.9 | 91.5 | 0.04 | 79.00 | 93 | 87 | 449 | 0.207 | 0.4 |
| RS096 | 1.14 | 15.5 | 25.81 | 19.4 | 174 | 11.4 | 1.2 | 53.9 | 0.05 | 119.56 | 97 | 87 | 452 | 0.194 | 0.5 |
| RS100 | 1.16 | 17.0 | 18.54 | 28.3 | 41 | 12.0 | 1.7 | 43.8 | 0.04 | 127.62 | 76 | 70 | 300 | 0.182 | <0.3 |
| RS106 | 1.44 | 48.8 | 103.51 | 95.3 | 119 | 27.1 | 9.1 | 63.4 | 0.17 | 252.69 | 107 | 87 | 481 | 0.239 | 0.6 |
| RS108 | 1.24 | 25.5 | 30.65 | 28.9 | 47 | 10.8 | 1.4 | 72.0 | 0.09 | 61.45 | 89 | 80 | 468 | 0.209 | <0.3 |
| RS112 | 0.65 | 52.5 | 125.76 | 43.9 | 163 | 13.7 | 3.2 | 313.1 | 0.08 | 1711.99 | 99 | 81 | 399 | 0.199 | <0.3 |
| RS115 | 1.48 | 33.4 | 25.04 | 33.4 | 173 | 11.4 | 2.7 | 147.0 | 0.06 | 118.07 | 118 | 101 | 502 | 0.234 | 0.7 |
| RS118 | 0.66 | 60.0 | 449.03 | 74.9 | 996 | 23.6 | 16.6 | 966.6 | 0.18 | >4000.00 | 117 | 109 | 524 | 0.202 | 0.6 |
| RS126 | 1.05 | 23.0 | 23.47 | 25.5 | 68 | 8.8 | 1.2 | 48.8 | 0.05 | 131.80 | 71 | 63 | 318 | 0.241 | <0.3 |
| RS128 | 1.03 | 12.5 | 15.16 | 14.5 | 59 | 8.1 | 1.6 | 33.6 | 0.03 | 99.13 | 83 | 71 | 295 | 0.204 | <0.3 |
| RS129 | 0.88 | 25.0 | 359.75 | 26.7 | 77 | 13.7 | 3.1 | 81.6 | <0.02 | 565.34 | 85 | 76 | 376 | 0.187 | 0.8 |
| RS131 | 0.12 | 19.0 | 228.86 | 34.7 | 679 | 13.4 | 3.8 | 895.5 | 0.10 | >4000.00 | 56 | 67 | 336 | 0.141 | 0.3 |
| RS134 | 1.24 | 34.2 | 37.56 | 30.1 | 103 | 20.2 | 6.7 | 91.1 | 0.03 | 151.98 | 115 | 102 | 555 | 0.290 | 0.7 |
| RS135 | 1.35 | 17.2 | 30.77 | 23.3 | 93 | 10.1 | 2.1 | 60.4 | 0.04 | 103.69 | 97 | 84 | 389 | 0.215 | 1.0 |
| RS143 | 0.23 | 29.2 | 221.68 | 41.1 | 651 | 15.9 | 6.4 | 671.9 | 0.14 | >4000.00 | 90 | 95 | 467 | 0.202 | <0.3 |
| RS144 | 1.34 | 17.9 | 25.37 | 20.1 | 50 | 12.3 | 1.9 | 47.3 | 0.04 | 217.65 | 111 | 95 | 505 | 0.227 | 1.2 |
| RS148 | 0.65 | 22.7 | 16.00 | 11.1 | 70 | 13.8 | 0.6 | 36.2 | 0.03 | 48.30 | 69 | 46 | 307 | 0.300 | <0.3 |
| RS151 | 1.48 | 26.9 | 12.26 | 16.6 | <20 | 17.0 | 0.8 | 37.6 | 0.03 | 45.82 | 139 | 84 | 606 | 0.372 | <0.3 |
| RS156 | 0.41 | 20.8 | 50.46 | 28.6 | 1164 | 8.1 | 1.6 | 361.7 | 0.10 | 3499.91 | 64 | 70 | 365 | 0.157 | 0.9 |
| RS159 | 0.95 | 12.8 | 15.37 | 11.3 | 57 | 7.6 | 0.7 | 31.0 | 0.03 | 69.45 | 72 | 50 | 280 | 0.262 | <0.3 |
| RS162 | 1.11 | 33.7 | 26.37 | 34.2 | 99 | 22.4 | 5.8 | 28.6 | 0.03 | 68.91 | 102 | 97 | 486 | 0.256 | 1.2 |
| RS164 | 1.41 | 36.5 | 19.59 | 31.3 | 112 | 17.7 | 5.9 | 24.6 | 0.04 | 79.33 | 113 | 95 | 512 | 0.193 | 1.2 |
| RS169 | 0.82 | 22.4 | 15.76 | 24.8 | 33 | 9.5 | 1.2 | 20.7 | 0.03 | 72.12 | 64 | 51 | 250 | 0.268 | <0.3 |
| Pulp Duplicates |  |  |  |  |  |  |  |  |  |  |  |  |  |  |  |
| SJ10 | 4.47 | 28.9 | 35.01 | 35.5 | 78 | 22.3 | 13.9 | 27.9 | 0.06 | 3.62 | 129 | 84 | 437 | 0.322 | 0.7 |
| SJ10 | 4.41 | 28.7 | 34.97 | 33.9 | 82 | 22.2 | 13.8 | 27.3 | 0.04 | 3.53 | 128 | 80 | 432 | 0.299 | 0.7 |
| RS090 | 1.10 | 22.8 | 22.39 | 25.0 | 37 | 15.5 | 1.7 | 76.7 | 0.06 | 101.96 | 120 | 88 | 520 | 0.314 | <0.3 |
| RS090 | 1.05 | 20.8 | 22.25 | 24.9 | 41 | 15.2 | 1.6 | 76.5 | 0.06 | 96.97 | 118 | 87 | 513 | 0.313 | <0.3 |
| RS037 | 1.38 | 19.0 | 47.29 | 49.8 | 110 | 19.5 | 2.5 | 1431.2 | <0.02 | 575.47 | 90 | 44 | 269 | 0.674 | 1.1 |
| RS037 | 1.36 | 19.5 | 48.43 | 50.8 | 107 | 19.6 | 2.5 | 1426.4 | 0.02 | 564.07 | 89 | 44 | 278 | 0.693 | 0.9 |
| Reference Materials |  |  |  |  |  |  |  |  |  |  |  |  |  |  |  |
| OREAS45H | 1.40 | 787.4 | 11.39 | 38.9 | 110 | 444.9 | 94.1 | 16.3 | 0.04 | 1.12 | 280 | 681 | 319 | 0.902 | 1.3 |
| OREAS501D | 93.39 | 2662.7 | 24.43 | 81.7 | 588 | 21.9 | 9.1 | 13.4 | 0.31 | 2.56 | 75 | 43 | 931 | 0.376 | 1.4 |
| OREAS45H | 1.46 | 800.2 | 11.49 | 39.3 | 129 | 450.7 | 94.9 | 16.1 | 0.03 | 0.84 | 287 | 708 | 322 | 0.928 | 1.4 |
| OREAS25A-4A | 2.37 | 33.1 | 23.32 | 43.7 | <20 | 47.9 | 7.9 | 10.0 | 0.06 | 5.50 | 164 | 119 | 146 | 0.954 | 2.2 |
| OREAS501D | 94.14 | 2651.9 | 24.30 | 83.2 | 639 | 21.6 | 8.9 | 11.4 | 0.24 | 2.43 | 69 | 42 | 958 | 0.364 | 1.9 |
| BLK | <0.05 | <0.1 | 0.21 | 0.2 | <20 | <0.1 | <0.2 | <0.2 | <0.02 | 0.21 | <2 | <1 | <1 | <0.001 | <0.3 |
| BLK | <0.05 | <0.1 | 0.12 | <0.2 | <20 | <0.1 | <0.2 | <0.2 | <0.02 | 0.08 | <2 | <1 | <1 | <0.001 | <0.3 |

**Appendix 2. Atomic Absortion Spectrometry of Mercury (AAS)**

The repeatability of the method was assessed based on 55 samples analysed between 25 and 26 January 2023, covering concentrations ranging from 20750 to 21.43 ppb. The samples were grouped into four concentration ranges. The mean RSD ranged from 12.23% to 0.05%.

Measurements were performed by Atomic Absorption Spectrometry of Mercury, using the Lumex RA-915 Lab instrument, whose high sensitivity and instrumental stability contribute to the precision observed in the results.

Most samples exhibited an RSD of less than 5%, meeting the acceptance criteria normally applied to instrumental methods. Only six samples exhibited an RSD above 7%, values justifiable by low concentration or intrinsic variability.

The certified standard NCS DC 73309, included in the series of measurements, presented a mean value of 75.60 ppb with an RSD of 1.73%, confirming the instrumental stability and the suitability of the method for quantification. The performance of this standard reinforces the reliability of the results obtained for the remaining samples, acting as an internal quality control and demonstrating that the method operates within the expected limits of precision.

Overall, the results confirm that the method exhibits good repeatability across the entire concentration range, making it suitable for routine use and for environmental monitoring purposes.

Table S2 - Hg (in ppb) determined by AAS in the soil samples from Ribeiro da Serra Mine.

| **Sample description** | **X mean (ppb)** | **SD** | **RSD %** | **N** |  |
| --- | --- | --- | --- | --- | --- |
| RS159 | 21.43 | 0.32 | 1.47 | 2 |  |
| RS151 | 30.76 | 0.38 | 1.24 | 2 |  |
| RS148 | 31.93 | 3.36 | 10.53 | 2 |  |
| RS112 | 34.86 | 1.2 | 3.44 | 2 |  |
| RS073 | 42.38 | 0.07 | 0.17 | 2 |  |
| RS055 | 43.2 | 0.94 | 2.18 | 2 |  |
| RS076 | 44.37 | 1.78 | 4.02 | 2 |  |
| RS004 | 44.38 | 0.24 | 0.55 | 2 |  |
| RS100 | 47.7 | 0.56 | 1.18 | 2 |  |
| RS067 | 49.63 | 0.73 | 1.48 | 2 |  |
| RS134 | 50.15 | 3.54 | 7.06 | 2 |  |
| RS084 | 53.82 | 0.53 | 0.99 | 2 |  |
| RS061 | 60.92 | 0.61 | 1 | 2 |  |
| RS094 | 71.28 | 4.17 | 5.85 | 2 |  |
| RS034 | 71.52 | 2.37 | 3.32 | 2 |  |
| RS088 | 71.89 | 0.36 | 0.51 | 2 |  |
| RS090 | 73.23 | 0.2 | 0.28 | 2 |  |
| **NCS DC 73309** | **75.6** | **1.34** | **1.77** | **2** |  |
| RS126 | 79.64 | 1.97 | 2.48 | 2 |  |
| RS066 | 82.82 | 2.28 | 2.75 | 2 |  |
| RS169 | 83.07 | 6.09 | 7.33 | 2 |  |
| RS049 | 84.12 | 6.1 | 7.25 | 2 |  |
| RS135 | 89.68 | 2.97 | 3.31 | 2 |  |
| RS128 | 92.77 | 2.52 | 2.72 | 2 |  |
| RS069 | 95.56 | 2.16 | 2.26 | 2 |  |
| RS108 | 105.9 | 2.63 | 2.49 | 2 |  |
| RS029 | 108.9 | 0.06 | 0.05 | 2 |  |
| RS164 | 110.8 | 0.31 | 0.28 | 2 |  |
| RS156 | 111.8 | 1.84 | 1.65 | 2 |  |
| RS037 | 114 | 4.79 | 4.2 | 2 |  |
| RS162 | 116 | 4.4 | 3.79 | 2 |  |
| RS051 | 125.4 | 3.31 | 2.64 | 2 |  |
| RS096 | 126.5 | 3.91 | 3.09 | 2 |  |
| RS045 | 126.9 | 5.38 | 4.24 | 2 |  |
| RS027 | 150 | 5.35 | 3.56 | 2 |  |
| RS144 | 160 | 0.97 | 0.61 | 2 |  |
| RS052 | 160.9 | 1.01 | 0.63 | 2 |  |
| RS057 | 180.7 | 2.23 | 1.24 | 2 |  |
| RS013 | 192.5 | 3.77 | 1.96 | 2 |  |
| RS115 | 198.2 | 0.3 | 0.15 | 2 |  |
| RS019 | 199.2 | 9.05 | 4.54 | 2 |  |
| RS021 | 225.8 | 0.61 | 0.27 | 2 |  |
| RS015 | 242.8 | 12.73 | 5.24 | 2 |  |
| RS017 | 247.2 | 0.18 | 0.07 | 2 |  |
| RS007 | 259.8 | 2.71 | 1.04 | 2 |  |
| RS023 | 268.1 | 4.72 | 1.76 | 2 |  |
| RS001 | 286 | 2.48 | 0.87 | 2 |  |
| RS106 | 324.2 | 12.57 | 3.88 | 2 |  |
| RS129 | 429.1 | 18.75 | 4.37 | 2 |  |
| RS040 | 607.8 | 1.73 | 0.29 | 2 |  |
| RS031 | 954.6 | 81.54 | 8.54 | 3 |  |
| RS143 | 1700 | 9.76 | 0.57 | 2 |  |
| RS078 | 2710 | 20.16 | 0.74 | 2 |  |
| RS118 | 9585 | 1172 | 12.23 | 3 |  |
| RS131 | 20750 | 865.9 | 4.17 | 3 |  |

**Appendix 3. Sequential Extraction of Mercury**

**Precision of the analytical result**

In this work, to estimate the relative intermediate precision associated to the Hg concentration in mine soil samples, eight samples (from 12) were randomly selected and submitted to the experimental procedure in duplicates to evaluate the pooled variability (Araujo 2009). Although working with all samples in duplicate would be advisable, this approach would be too laborious and time-consuming.

It is important to mention that the precision obtained by this approach englobes all sources of error from the procedure as a whole and not only the precision of the instrumental analysis step. The obtained value was adopted as characteristic of the complete analytical procedure and, thus, was considered applicable (homogeneity of variance) to the whole concentration range found in the samples.

The precision value was obtained through the following equation:

$S_{rel}= \sqrt{\frac{\frac{\left( {y_{1}-y}_{1}^{'} \right)^{2}}{2\bar{y}_{1}^{2}} + \frac{\left( {y_{2}-y}_{2}^{'} \right)^{2}}{2\bar{y}_{2}^{2}} + \ldots+ \frac{\left( {y_{n}-y}_{n}^{'} \right)^{2}}{2\bar{y}_{n}^{2}}}{n}}$ (Eq. S1)

Where:

$\bar{y_{i}}= \frac{y_{i}+ y_{i}^{'}}{2}$ ,

and *y_n_* and *y’_n_* are the values obtained for a sample, and it’s replicate, respectively, and *n* is the number of samples replicated (n = 8).

**Certified reference material (CRM) analysis**

A CRM - [RTC CRM005: Trace Metals - sewage amended soil] was used to assess the performance of the sequential extraction procedure (SEP) US EPA Method 3200. The CRM was submitted to the same SEP in duplicate alongside the samples. This material is certified for total mercury concentration.

The pseudo-total Hg concentration in the CRM was determined by subjecting the material to microwave-assisted digestion with aqua regia according to USEPA 3051 method to assess the accuracy and precision of the analytical determination of mercury independently of the sequential extraction procedure. This analysis was carried out in five replicates (Table S1).

The recovery percentage ranged from 86 to 118% with a mean value of 97% ± 11% relative to the certified value, indicating that the analytical method for Hg determination was efficient.

Table S3 - Recovery percentage of Hg in the CRM005 obtained by microwave digestion using aqua regia.

| **CRM digestion** | | | |
| --- | --- | --- | --- |
| **Assay** | **certified value / mg.kg^-1^** | **measured [Hg] / mg.kg^-1^** | **Recovery / %** |
| 1 | 3,22 ± 0.134  (3.35 - 3.08) | 3,1 | 95 |
| 2 |  | 3,8 | 118 |
| 3 |  | 2,8 | 88 |
| 4 |  | 3,2 | 99 |
| 5 |  | 2,8 | 86 |
|  | **Mean** | **3,1** | **97** |
|  | **SD** | **0,4** | **11** |

Additionally, the cumulative concentrations obtained and corresponding percentages relative to the certified value, are presented in Table S2.

Table S4 - Recovery percentage of Hg in the CRM005 obtained by the SEP.

| **CRM sequential extraction** | | | |
| --- | --- | --- | --- |
| **Assay** | **certified value / mg.kg^-1^** | **[Hg]_sum of fractions_ / mg.kg^-1^** | **Recovery / %** |
| 1 | 3,22 ± 0.134 | 2,07 | 64 |
| 2 | (3.35 - 3.08) | 1,48 | 46 |
|  | **Mean** | **1,8** | **55** |
|  | **SD** | **0,4** | **13** |

The discrepancy observed between the total mercury content and the cumulative recovery obtained through the SEP can be attributed to the operational nature of the SEP method (employing weaker solutions) and the strong association of mercury with stable phases in the sewage-amended soil matrix, rather than to analytical errors (Breslin 1999).

**Appendix 4. Total Organic Carbon (TOC)**

Measurements were calibrated using the IFP 160000 standard. The concentrations of total organic carbon (TOC) and mineral carbon (MinC) are associated with uncertainties of ±0.01 wt.% based on the IFP 160000 calibration standard (Vinci Technologies, France), which has a total organic carbon content of 3.28 ± 0.14 wt.%..

Table 5 - Total Organic Carbon determined in the soils surrounding Ribeiro da Serra Mine.

| **Sample** | **PC [%]** | **RC [%]** | **TOC [%]** | **MINC [%]** | **HI [mg HC/g TOC]** | **OI [mg CO2/g TOC]** | **Tmax [°C]** | **S1 [mg HC/g]** | **S2a [mg HC/g]** | **S2b [mg HC/g]** | **S3** |  |  |
| --- | --- | --- | --- | --- | --- | --- | --- | --- | --- | --- | --- | --- | --- |
| IFP160000 | 1.06 | 2.22 | 3.28 | 3.26 | 379 | 24 | 416 | 0.02 | 12.43 | 0.00 | 0.79 |  |  |
| RS002 | 0.54 | 2.93 | 3.47 | 0.33 | 134 | 162 | 412 | 0.06 | 4.65 | 0.00 | 5.64 |  |  |
| RS004 | 0.48 | 2.18 | 2.67 | 0.22 | 160 | 169 | 414 | 0.04 | 4.27 | 0.00 | 4.50 |  |  |
| RS005 | 0.49 | 2.83 | 3.32 | 0.24 | 130 | 142 | 417 | 0.03 | 4.32 | 0.00 | 4.71 |  |  |
| RS008 | 0.93 | 4.94 | 5.87 | 0.32 | 149 | 120 | 416 | 0.08 | 8.76 | 0.00 | 7.06 |  |  |
| RS022 | 0.23 | 1.54 | 1.76 | 0.23 | 90 | 186 | 379 | 0.06 | 1.58 | 0.00 | 3.27 |  |  |
| RS023 | 0.29 | 2.08 | 2.37 | 0.28 | 91 | 161 | 393 | 0.03 | 2.16 | 0.00 | 3.80 |  |  |
| RS024 | 0.51 | 2.95 | 3.46 | 0.24 | 129 | 146 | 412 | 0.04 | 4.46 | 0.00 | 5.05 |  |  |
|  | 0.00 | 0.00 | 0.00 | 0.00 |  |  | -2 | 0.00 | 0.00 | 0.00 | 0.00 |  |  |
| RS025 | 0.83 | 3.55 | 4.38 | 0.29 | 179 | 148 | 418 | 0.06 | 7.85 | 0.00 | 6.50 |  |  |
| RS026 | 0.67 | 3.67 | 4.34 | 0.50 | 132 | 155 | 400 | 0.09 | 5.73 | 0.00 | 6.72 |  |  |
| RS029 | 0.51 | 3.13 | 3.64 | 0.21 | 123 | 138 | 418 | 0.02 | 4.47 | 0.00 | 5.03 |  |  |
| RS031 | 0.56 | 3.73 | 4.29 | 0.31 | 112 | 139 | 410 | 0.05 | 4.79 | 0.00 | 5.98 |  |  |
| RS034 | 0.48 | 3.02 | 3.50 | 0.28 | 109 | 174 | 414 | 0.02 | 3.82 | 0.00 | 6.09 |  |  |
| RS035 | 0.93 | 5.40 | 6.33 | 0.41 | 130 | 136 | 410 | 0.14 | 8.25 | 0.00 | 8.60 |  |  |
| RS040 | 0.36 | 2.49 | 2.86 | 0.28 | 88 | 197 | 405 | 0.01 | 2.52 | 0.00 | 5.61 |  |  |
| RS043 | 0.35 | 2.44 | 2.79 | 0.23 | 82 | 201 | 393 | 0.03 | 2.29 | 0.00 | 5.60 | | |
| RS044 | 0.17 | 0.88 | 1.05 | 0.23 | 107 | 246 | 383 | 0.08 | 1.11 | 0.00 | 2.57 | |  |
| RS051 | 0.83 | 4.23 | 5.06 | 0.42 | 149 | 146 | 415 | 0.04 | 7.52 | 0.00 | 7.37 | |  |
| RS053 | 0.12 | 0.68 | 0.79 | 0.25 | 74 | 324 | 392 | 0.01 | 0.59 | 0.00 | 2.58 | |  |
| RS056 | 0.24 | 1.64 | 1.89 | 0.25 | 83 | 216 | 386 | 0.02 | 1.56 | 0.00 | 4.08 | |  |
| RS073 | 0.49 | 2.27 | 2.76 | 0.25 | 154 | 181 | 414 | 0.03 | 4.26 | 0.00 | 5.02 | |  |
| RS076 | 0.58 | 3.49 | 4.08 | 0.35 | 110 | 190 | 408 | 0.02 | 4.48 | 0.00 | 7.74 | |  |
| RS078 | 0.11 | 0.38 | 0.49 | 0.41 | 71 | 611 | 334 | 0.01 | 0.35 | 0.00 | 2.98 | |  |
| RS084 | 0.84 | 3.39 | 4.23 | 0.26 | 181 | 176 | 418 | 0.05 | 7.64 | 0.00 | 7.43 | |  |
| RS091 | 0.29 | 1.79 | 2.08 | 0.79 | 98 | 210 | 380 | 0.02 | 2.05 | 0.00 | 4.35 | |  |
| RS093 | 0.58 | 3.10 | 3.67 | 0.25 | 138 | 154 | 408 | 0.04 | 5.08 | 0.00 | 5.65 | |  |
| RS094 | 0.63 | 3.05 | 3.68 | 0.32 | 149 | 170 | 418 | 0.02 | 5.49 | 0.00 | 6.25 | |  |
| RS097 | 0.11 | 0.63 | 0.75 | 0.17 | 72 | 336 | 391 | 0.01 | 0.54 | 0.00 | 2.51 | |  |
| RS099 | 0.46 | 2.36 | 2.82 | 0.26 | 137 | 182 | 397 | 0.04 | 3.85 | 0.00 | 5.12 | |  |
| RS105 | 0.50 | 17.34 | 17.84 | 0.39 | 23 | 32 | 424 | 0.01 | 4.17 | 0.00 | 5.64 | |  |
| RS110 | 1.63 | 6.26 | 7.89 | 0.52 | 204 | 134 | 421 | 0.06 | 16.10 | 0.00 | 10.58 | |  |
| RS112 | 0.16 | 0.85 | 1.02 | 0.18 | 95 | 295 | 378 | 0.02 | 0.96 | 0.00 | 3.00 | |  |
| RS118 | 0.37 | 2.18 | 2.54 | 0.68 | 80 | 286 | 389 | 0.01 | 2.04 | 0.00 | 7.27 | |  |
| RS119 | 0.34 | 6.12 | 6.46 | 0.48 | 34 | 88 | 412 | 0.01 | 2.20 | 0.00 | 5.69 | |  |
| RS129 | 0.92 | 5.02 | 5.95 | 0.37 | 137 | 152 | 415 | 0.03 | 8.14 | 0.00 | 9.03 | |  |
| RS131 | 0.27 | 1.48 | 1.75 | 0.33 | 89 | 294 | 394 | 0.00 | 1.56 | 0.00 | 5.16 | |  |
| RS132 | 0.31 | 1.86 | 2.17 | 0.51 | 114 | 172 | 387 | 0.04 | 2.48 | 0.00 | 3.73 | |  |
| RS135 | 1.31 | 6.18 | 7.49 | 0.45 | 157 | 160 | 423 | 0.03 | 11.78 | 0.00 | 11.99 | |  |
| RS137 | 0.42 | 1.37 | 1.78 | 0.17 | 224 | 160 | 410 | 0.10 | 4.00 | 0.00 | 2.86 | |  |
| RS143 | 0.41 | 2.49 | 2.90 | 0.51 | 96 | 225 | 384 | 0.01 | 2.80 | 0.00 | 6.53 | |  |
| RS144 | 0.81 | 4.32 | 5.13 | 0.39 | 132 | 176 | 418 | 0.04 | 6.77 | 0.00 | 9.06 | |  |
| RS148 | 0.25 | 0.97 | 1.22 | 0.20 | 180 | 208 | 277 | 0.02 | 2.20 | 0.00 | 2.54 | |  |
| RS158 | 0.26 | 1.38 | 1.64 | 0.12 | 130 | 179 | 405 | 0.02 | 2.13 | 0.00 | 2.93 | |  |
| RS159 | 0.22 | 0.92 | 1.14 | 0.15 | 168 | 192 | 411 | 0.02 | 1.91 | 0.00 | 2.19 | |  |
| RS171 | 1.22 | 5.36 | 6.58 | 0.40 | 172 | 156 | 418 | 0.05 | 11.32 | 0.00 | 10.27 | |  |
| IFP160000 | 1.06 | 2.22 | 3.28 | 3.26 | 379 | 24 | 416 | 0.02 | 12.43 | 0.00 | 0.79 | |  |

**References**

Araujo, P. Key Aspects of Analytical Method Validation and Linearity Evaluation. *Journal of Chromatography B: Analytical Technologies in the Biomedical and Life Sciences* **2009**, *877* (23), 2224–2234. https://doi.org/10.1016/j.jchromb.2008.09.030.

Breslin, V. T. Retention of Metals in Agricultural Soils After Amending with MSW and MSW-Biosolids Compost. *Water Air Soil Pollut.* **1999**, *109* (1–4), 163–178. https://doi.org/10.1023/A:1005026131978.
